# Alveolarization Genes Modulated by Fetal Tracheal Occlusion in the Rabbit Model for Congenital Diaphragmatic Hernia: A Randomized Study

**DOI:** 10.1371/journal.pone.0069210

**Published:** 2013-07-01

**Authors:** Aline Vuckovic, Susanne Herber-Jonat, Andreas W. Flemmer, Xenia I. Roubliova, Jacques C. Jani

**Affiliations:** 1 Laboratory of Physiology and Physiopathology, Faculty of Medicine, Université Libre de Bruxelles, Brussels, Belgium; 2 Division of Neonatology, University Children’s Hospital, Perinatal Center, Ludwig-Maximilian-University Munich, Großhadern, Munich, Germany; 3 HistoGeneX, AZ-Middelheim, Antwerp, Belgium; 4 Department of Obstetrics and Gynecology, University Hospital Brugmann, Brussels, Belgium; University of Giessen Lung Center, Germany

## Abstract

**Background:**

The mechanisms by which tracheal occlusion (TO) improves alveolarization in congenital diaphragmatic hernia (CDH) are incompletely understood. Therefore transcriptional and histological effects of TO on alveolarization were studied in the rabbit model for CDH. The question of the best normalization strategy for gene expression analysis was also addressed.

**Methods:**

Fetal rabbits were randomized for CDH or sham operation on gestational day 23/31 and for TO or sham operation on day 28/31 resulting in four study groups. Untouched littermates were added. At term and before lung harvest, fetuses were subjected to mechanical ventilation or not. Quantitative real-time PCR was performed on lungs from 4–5 fetuses of each group with and without previous ventilation. Stability of ten housekeeping genes (HKGs) and optimal number of HKGs for normalization were determined, followed by assessment of HKG expression levels. Expression levels of eleven target genes were studied in ventilated lungs, including genes regulating elastogenesis, cell-environment interactions, and thinning of alveolar walls. Elastic staining, immunohistochemistry and Western blotting completed gene analysis.

**Results:**

Regarding HKG expression, TO increased β-actin and β-subunit of ATP synthase. Mechanical ventilation increased β-actin and β2-microglobulin. Flavoprotein subunit of succinate dehydrogenase and DNA topoisomerase were the most stable HKGs. CDH lungs showed disorganized elastin deposition with lower levels for tropoelastin, fibulin-5, tenascin-C, and α6-integrin. After TO, CDH lungs displayed a normal pattern of elastin distribution with increased levels for tropoelastin, fibulin-5, tenascin-C, α6-integrin, ß1-integrin, lysyl oxidase, and drebrin. TO increased transcription and immunoreactivity of tissue inhibitor of metalloproteinase-1.

**Conclusions:**

Experimental TO might improve alveolarization through the mechanoregulation of crucial genes for late lung development. However part of the transcriptional changes involved genes that were not affected in CDH, raising the question of TO-induced disturbances of alveolar remodeling. Attention should also be paid to selection of HKGs for studies on mechanotransduction-mediated gene expressions.

## Introduction

Among the physical forces exerting on the lung during gestation, internal pressures due to fluid secretion across the alveolar epithelium into the airways in formation are crucial for normal pulmonary development [1]. The role of intraluminal pressures in maintaining proper lung expansion has been highlighted by experiments using fetal tracheal ligation or occlusion (TO). Preventing the efflux of fluid out of the lungs, TO increases intraluminal pressures and tissue stretch leading to accelerated lung growth and maturation [2,3]. Since the first reports in the late 1960’s, TO has been used to understand the mechanisms of lung growth induced by mechanotransduction [3], as well as to remedy lung hypoplasia with special consideration to congenital diaphragmatic hernia (CDH) [2,4,5]. In animal models of CDH, TO improves airway branching, vascular development, and alveolar formation [4–6]. As such, clinical TO has been developed to improve the outcome of human fetuses with severe CDH [7].

In vitro studies focusing on the connections between lung stretch and induced cellular mechanisms have shown that mechanical forces stimulate mechanoreceptors in pulmonary cells, activate signaling pathways, and modify the transcriptional activity of several genes [8,9]. This suggests that TO-mediated mechanotransduction might induce changes in the expression of genes involved in lung development and maturation. Therefore recent attention has been drawn to transcriptional changes occurring in intact and/or hypoplastic lungs after TO in sheep and rodents, including genes encoding for growth factors [6,10–12], transcription factors [13], cell cycle proteins [10,13,14], metabolic enzymes [14], epithelial markers [10,13,15], ion channels [10], vasoactive mediators [16], and extracellular matrix (ECM) molecules [6,13].

The creation of a large gas exchange surface area during the alveolar development is the pivotal step for a successful adaptation to extrauterine life. This process is characterized by formation of septa guided by elastic fibers to produce alveoli, thinning of alveolar walls, and expansion of the capillary network underlining the alveolar epithelium. The molecular mechanisms controlling alveolarization involve various growth factor signalings, transcriptions factors, and ECM components [17]. In human and experimental CDH, defective alveolarization has been linked to abnormal growth factors signalings, leading to inadequate elastin synthesis and deposition as well as vascular underdevelopment [6,18–21]. Although the enhancement of alveolar formation is one of the most striking known effects of TO, the transcriptional mechanisms by which TO stimulates alveolarization have not been completely elucidated. Most previous reports have indeed focused on genes encoding for growth factors [6,22,23] with less attention paid to ECM, elastin excepted [6,24–26]. However the effect of TO on the matrix environment might be of particular interest since TO rapidly induces a myofibroblast phenotype during the pre-alveolar stage of lung development [14].

The surgically induced CDH model in the fetal rabbit directly impacts on physical factors exerting on the developing lung by compression of the ipsilateral lung and interference with fetal breathing movements, suppressing therefore cyclic fluctuation of intratracheal pressure during fetal life. This model recapitulates most histological, functional and biological features of human CDH lungs [19,27,28]. Further, it is of particular interest to study alveolar formation since unlike sheep [29] and rodents [30] the rabbit shares close similarities with the human alveolar development [31]. This model has been formerly used to determine the effects of TO at morphological [4,5] and functional levels [32] with much less emphasis on molecular events.

The main objective of the present work was to assess the consequences of TO on alveolarization in the rabbit model for CDH, based on histological evaluation and gene expression analysis by quantitative real-time PCR (qPCR). We focused on alveolarization-relevant genes related to the ECM, such as genes encoding for matrix components (including those controlling synthesis, assembly, and stability of elastic fibers), regulating interactions between cells and their extracellular environment, or participating in the remodeling of alveolar walls. We hypothesized that the transcription of those genes might be affected by changes in mechanical forces in case of pulmonary hypoplasia and/or overdistension by TO. Transcriptional variations were also confronted with protein expression changes assessed by immunohistochemistry and Western blot analysis. In addition we addressed methodological questions related to the use of the rabbit model in the present work and for future studies. As an important pre-requisite for gene expression analysis, the most appropriate combination of housekeeping genes (HKGs) for normalization of qPCR data was determined in the rabbit fetus under various experimental set-ups of interest, including sham operation, surgically induced CDH, TO, and mechanical ventilation. Finally, the most reliable control group was identified by comparisons between non-operated and sham-operated fetuses.

## Methods

### Animals and ethics Statement

Forty-three rabbit fetuses were obtained from 2 ongoing sets of experiments aiming at evaluating lung biology after TO in the surgically induced CDH model. Thirty-seven pregnant New Zealand White female rabbits obtained at 15 days’ gestational age (GA) were housed as previously reported [19,32]. The offspring of the first set of 19 does (obtained from CEGAV, France) was euthanatized just before delivery to investigate biological changes before adaptation to extrauterine life occurs. The offspring of the second set of 18 does (obtained from Charles River, Germany) was mechanically ventilated before the lung tissue was processed for further investigation. The protocols were approved by the Institutional Ethics Committee of the “Université Libre de Bruxelles” (Brussels, Belgium, number 321N) and the Bavarian Animal Research Authority (Munich, Germany, number 55.2-1-54-2531-149-08). All experiments were carried out in agreement with the guidelines of laboratory animals’ care and use of the U.S. National Institutes of Health.

### Experimental protocols and tissue collection

**Figure 1:**
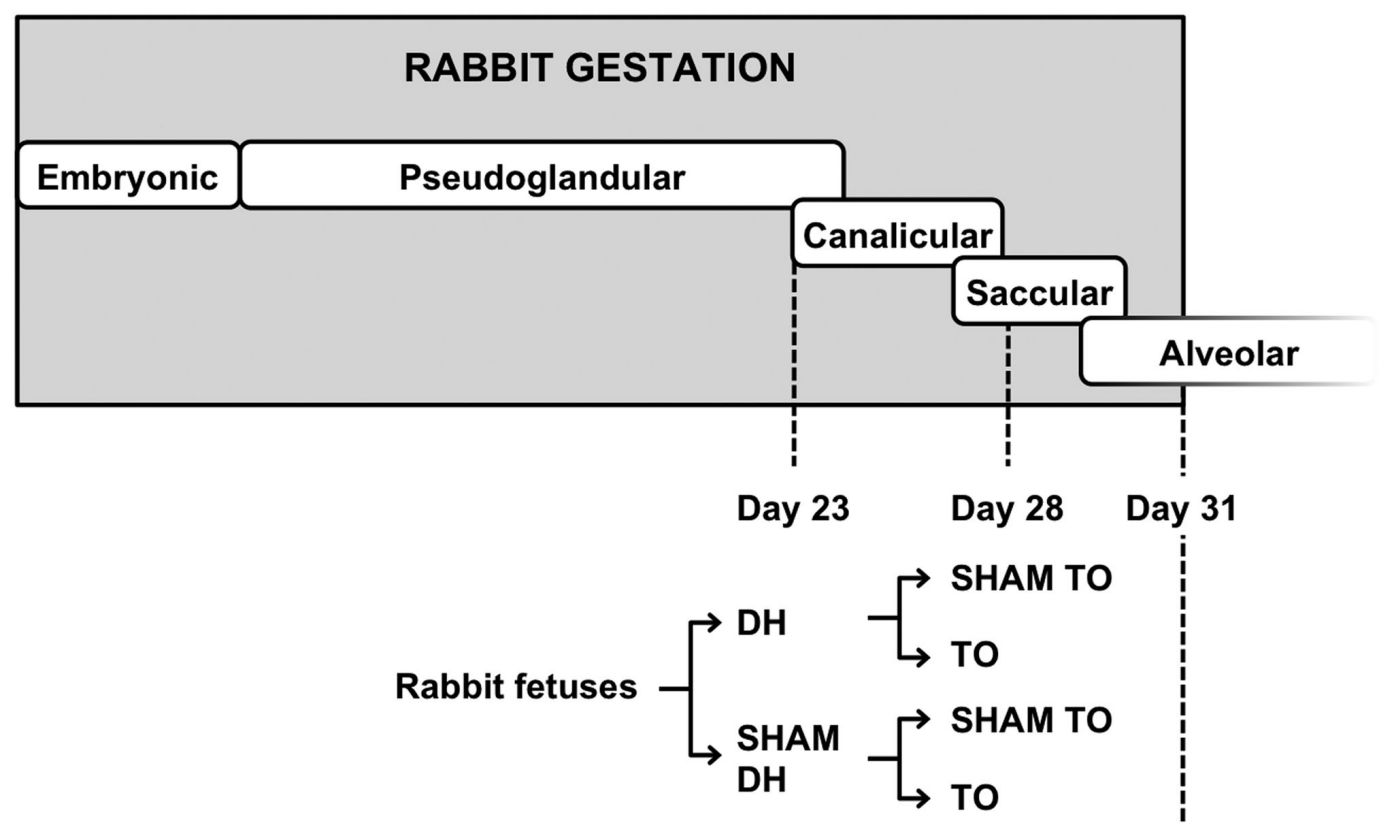
Experimental design in the rabbit fetus. Fetal rabbits underwent two successive randomized surgeries during the late pulmonary development (at 23 and 28 days’ gestational age). In the first set of experiments, fetal lungs were directly harvested at term without postnatal experiments. In the second set of experiments, fetuses were ventilated at term before tissue sampling. SHAM, sham-operated fetuses; DH, diaphragmatic hernia fetuses; DH+ TO, DH fetuses with tracheal occlusion; TO, sham DH fetuses with TO.

Anesthesia and surgical procedures have been described elsewhere [4,19,28,32]. Fetuses located at the ovarian end of each uterine horn were selected, since they do not exhibit fetal growth retardation as compared to fetuses situated near the vaginal end [33]. The experimental protocol was based on a 2x2 factorial design. Fetuses were assigned to each type of surgery (neither intervention, one or the other, or both) using a computer-generated random allocation sequence and a block randomization ensuring similar number of subjects in each group (http:/www.randomization.com/). Briefly, at 23 days’ GA (pseudoglandular stage; Figure 1), 2 fetuses per doe were assigned to a partial excision of the diaphragm through a left thoracotomy (DH) or a thoracotomy without diaphragmatic incision (sham DH). Five days later (late canalicular stage), the previously operated fetuses were subjected to a tracheal ligation (TO) or a tissue dissection (sham TO). Based on this protocol four study groups were defined: SHAM (sham DH/sham TO), TO (sham DH/TO), DH (DH/sham TO), and DH+ TO (DH/TO). Non-operated fetuses were added for comparisons with SHAM fetuses. At term (31 days’ GA), living operated fetuses and non-operated controls were delivered by cesarean section. Maternal anesthesia and parameters were closely monitored during fetal harvesting. After cesarean section each doe was killed with an overdose of sodium pentobarbital. In the first set of experiments, fetuses were euthanatized during cesarean section with an overdose of anesthetics to avoid pulmonary changes occurring at and after birth. In the second set of experiments, fetuses were delivered, anesthetized, and ventilated during 30 minutes to assess lung mechanics before euthanasia [32]. Ventilation was maintained with 21% O2 with physiological breathing rate (120 breaths/minute), low tidal volume (8 ml/kg), and low positive end-expiratory pressure (3 cm H_2_O), conditions that would unlikely alter profoundly the pulmonary parenchyma or induce severe inflammatory processes [34,35]. Ventilation data and repeated lung function measurements are not reported in this work, as the sample size needed to evidence significant differences between groups in terms of lung mechanics (*n* = 8 to 10) has not been reached yet at the time of the completion of the current study. Fetal lungs were harvested and weighed for the calculation of the lung to body weight ratio (LBWR). The left lung was immediately snap frozen in liquid nitrogen and stored at −80°C until RNA and protein extraction. The right lower lobe was kept at −80°C for future biological studies. The right upper lobe of previous ventilated fetuses was fixed in a device filled with paraformaldehyde and subjected overnight to a low vacuum in order to avoid tissue collapse [36]. In the absence of previous qPCR data in the fetal rabbit lung undergoing TO, sample size was estimated by the sample maximization strategy for qPCR experiments [37] taking into account previous qPCR reports [38]. Laboratory experiments were thus started as soon as 4 to 5 fetuses per group were obtained. All samples (*n* = 43) were included in a pilot analysis for the selection of optimal HKGs under various conditions. Lungs from ventilated fetuses (*n* = 22) were used to study the effects of TO on alveolarization at transcriptional, translational, and histological level.

### Elastin quantification on lung tissue section

After deparaffinization in xylene and rehydration through graded ethanol, the slides (5 µm, transverse cut) were oxidized with potassium permanganate and decolorized with oxalic acid. Subsequently, the slides were rinsed with water, and Miller’s stain was applied. The sections were washed and successively treated with Celestin Blue, hematoxylin, and Curtis’ stain before dehydration and mounting. Sections were analyzed using a light microscope connected to a digital camera (CarlZeiss, Oberkochen, Germany). Eight random non-overlapping fields per lung were captured in color at a magnification of x40. For each lung the whole pulmonary section was considered so that any lung heterogeneity could be taken into account in the final calculations. Two blinded observers (AV and XR) used the point-counting method to measure elastin deposits at the tips of secondary crests, elastin fibers within the alveolar walls, and the percentage of lung occupied by tissue. The number of times the point grid fell on elastin-containing crests was expressed as a percentage of tissue area. The number of times a dark elastic fiber fell on the grid was related to the number of times alveolar walls fell on the grid.

### RNA extraction and reverse transcription

Total RNA from whole frozen lungs (100 mg) was isolated using Trizol reagent (Life Technologies, Ghent, Belgium) as previously described [19]. The RNA concentration and the ratio of absorbance at 260 nm to 280 nm and 260 nm to 230 nm were measured by optical density (NanoDrop 2000, Thermo Scientific, Wilmington, DE). RNA integrity was confirmed by 1.5% agarose gel electrophoresis. The absence of genomic DNA was verified by qPCR performed on total RNA that has not been reverse transcribed as a template. RNA concentrations were adjusted at 1 µg/µl in nuclease-free water, and stored at −80°C. The day after RNA isolation, reverse transcription with random hexamers was performed with 2 µg of total RNA in a 20-µl total reaction volume following the manufacturer’s instructions (Life Technologies) [19]. The cDNA obtained was stored at −20°C until use.

### Primer design and validation

We previously reported in the fetal rabbit model for CDH primer pairs for target genes involved in alveolarization (tropoelastin, ELN; lysyl oxidase, LOX; fibulin-5, FBLN5; tenascin-C, TNC; drebrin, DBN1), as well as for ten candidate HKGs that belong to different functional classes and are constitutively expressed in the lung (Supporting Information; Table S1-S3) [19]. The *Primer3* program (http://frodo.wi.mit.edu/primer3/) was used to generate new primers for α6- and ß1-integrin subunits (ITGA6 and ITGB1), metalloproteinases (MMPs; MMP2 and MMP14), and tissue inhibitors of MMPs (TIMPs; TIMP1 and TIMP2). Because of few specific nucleic acid sequences known in the rabbit, predicted rabbit sequences produced by the NCBI’s Genome Annotation Project or reviewed human sequences from GenBank were also considered (Table 1). A *BLAST* analysis against the whole database (http://www.ncbi.nlm.nih.gov/BLAST/) was run to check if primer pairs were matching at the sequence of interest and at similar sequences from other species. The absence of secondary structures was verified using the Mfold program (http://mfold.rna.albany.edu/). Oligonucleotides were synthesized at Eurogentec (Liège, Belgium). PCR efficiencies in the exponential phase were calculated by standard curves run in triplicate (7 dilution series), according to the equation E = 10 [-1/slope] [37]. The generation of a single specific amplicon was confirmed by melting curve analysis and 2.5% agarose gel electrophoresis to maximize specific gene recognition and qPCR amplification [19]. Details about BLAST analysis, melting curve analysis, and gel electrophoresis regarding the newly designed primers were included in the Supporting Information (Text S1 Figure S1-S2).

**Table 1:** Sequences of primers newly designed for qPCR analysis.

**Gene (accession number)**	**Primer sequences - 5’ to 3’**	**T_m_ (°C)**	**E**	**R^2^**
ITGA6 (XM_002712183)	F: GTGACTGTGTTTCCCTCCAAG	59.61	1.995	0.997
	R: GCAAGCATCAAAATCCCAAC	60.46		
	Amplicon size: 96 bp			
ITGB1 (XM_002721189)	F: TGTAATGGCCGGGGTATCT	60.16	2.095	0.998
	R: TCTGGCACATCTCACAGGTT	59.26		
	Amplicon size: 85 bp			
MMP2 (NM_001082209)	F: CCCCAAAACGGACAAAGAG	60.47	2.033	0.999
	R: TCCTTCAGCACGAACAGGT	59.40		
	Amplicon size: 90 bp			
MMP14 (NM_004995)	F: CGAGGGGAGATGTTTGTCTT	59.14	1.907	0.998
	R: TCGTAGGCAGTGTTGATGGA	60.26		
	Amplicon size: 131 bp			
TIMP1 (NM_001082232)	F: ACTGAAGGCTGCTCCTGTTG	60.59	1.988	0.998
	R: GTGTGGGACAAAGAAAGATGG	59.44		
	Amplicon size: 80 bp			
TIMP2 (AF_069713)	F: CGACAAGGACATCGAGTTCA	59.83		
	R: GCCTTCCCTGCAATGAGATA	60.18	2.000	0.999
	Amplicon size: 102 bp			

F, forward primer; R, reverse primer; T_m_, melting temperature; bp, number of base pairs; E, real-time PCR efficiency; R^2^, coefficient of determination

### Quantitative real-time PCR

SYBR Green qPCR analysis was carried out with the Icycler iQ detection system (Bio-Rad Laboratories, Hemel Hempstead, UK). Procedure and amplification protocols have been previously reported [19]. For a given gene, all samples were analyzed in the same qPCR run according to the sample maximization strategy to minimize technical variations and ensure reliable estimation of gene expression levels [39]. For the analysis of HKG expression stability, the 43 lung samples were therefore measured in duplicates, whereas the analysis of target gene expressions was performed in 22 ventilated lung samples measured in triplicates. At the end of each run, the software used the recorded fluorescence measurements to calculate the quantification cycle (Cq) value for each well, which was defined by the number of PCR cycles at which fluorescence increases significantly above the background. To ensure the quality of measurements, negative (no template) and positive controls were included. In no template controls, amplification was not detected or Cq values were above 36. Intra-assay variation expressed as standard deviation of the Cq variance was between 0.03 and 0.09.

### Determination of housekeeping gene expression stability

Raw Cq data for the ten candidate HKGs were analyzed with the *genorm* software (http://www.biogazelle.com/) [39,40]. Firstly *genorm* established the expression stability of the candidate HKGs, which is defined as the constancy of the level of expression of a particular housekeeping gene in a given cell type, organ or experiment. The most stable gene identified by *genorm* among the other candidate genes exhibits therefore a level of expression that is less affected by experimental conditions than the other genes. For each experimental set-up, candidate HKGs were ranked based on the calculation of a gene expression stability parameter (M), which is the mean pair-wise variation for a gene from all other tested genes [40]. At each step, the gene with the highest M value (i.e. the less stable gene) was excluded for next analysis and the M values were recalculated until the most stable gene remained. An M value below 0.5 indicated a stably expressed gene. Besides M values, the software secondly provided additional data by calculating the coefficient of variation (CV) of the normalized gene relative quantities. A CV value below 0.2 was considered as acceptable [39]. The optimal number of HKGs was secondly determined by V analysis comparing the magnitude of the change in the normalization factor (NFn) after the inclusion of an additional HKG (NFn+1). The Vn/n+1 cut-off value of 0.15 indicated a set of stably expressed HKGs [40].

### Analysis and normalization of gene expression data

Because Cq values are logarithmic, they underestimate the true degree of variation that would be obvious in case of linear conversion. For each HKG and target gene, averaged Cq values obtained for each sample were therefore converted to linear values (Q) according to the efficiency corrected model [41]: Q = E^ΔCq^, where E is the efficiency of PCR reaction and ΔCq is the difference between the averaged Cq value of a sample and that of the sample with the highest expression (minimum Cq value) in the data set. To obtain normalized gene expression values, linear values were then divided by a normalization factor that was the geometric mean of the linear values calculated for the combination of the most stable HKGs identified by *genorm* [40]. Normalized expression levels obtained for each HKG were compared to evaluate the impact of mechanical ventilation and surgical procedures (sham operation, diaphragmatic excision, and tracheal ligation). Normalized expression levels of target genes were compared to assess the impact of TO on intact and hypoplastic lungs.

### MIQE Guidelines

The Minimum Information for Publication of Quantitative Real-Time PCR Experiments (MIQE) guidelines [37] were respected to improve the transparency and reliability of qPCR experiments (MIQE checklist detailed in the Supporting Information; Table S4).

### Immunohistochemical detection of MMPs and TIMPs

Immunohistochemistry was based on the avidin-biotin peroxidase method. After deparaffinization and rehydration, the slides were immersed in 1% hydrogen peroxide in tris-buffered saline (TBS) for 30 minutes at room temperature to quench endogenous peroxidase activity. Subsequently non-specific antigenic sites were blocked with 5% normal goat or mouse serum in TBS (Dako, Heverlee, Belgium) for 1 hour at room temperature. Sections were then incubated overnight at 4°C with the following primary antibodies diluted in TBS: mouse monoclonal antibody against the C-terminal region of MMP2 (clone 42-5D11, Merck Millipore, Overijse, Belgium; 1:250), mouse monoclonal antibody against the hemopexin-like domain of MMP14 (clone 113-5B7, abcam, Cambridge, UK; 1:25), goat polyclonal antibody against the C-terminal region of TIMP1 (sc-6832, Santa Cruz, Heidelberg, Germany; 1:50), and mouse monoclonal antibody against the N-terminal region of TIMP2 (clone 3A4, abcam; 1:50). Negative controls were realized by omission of the primary antibody. Primary antibodies have been confirmed to cross-react with rabbit tissue [42–44]. Subsequently the sections were washed with TBS and treated for 30 minutes at room temperature either with undiluted EnVision+ HRP complex (Dako), or with mouse anti-goat biotinylated secondary antibody (sc-53799, Santa Cruz; 1:200), followed by Vectastain ABC kit reagents according to the manufacturer’s instructions (Vector Laboratories, Burlingame, CA). After rinsing in TBS and incubation with a diaminobenzidine solution (Dako), sections were counterstained with hematoxylin, dehydrated through graded isopropanol, cleared in toluene, and mounted with DPX media (VWR, Leuven, Belgium). Color pictures were captured at a magnification of x40 using a light microscope connected to a digital camera (CarlZeiss). Immunohistochemistry was performed twice to ensure reproducibility.

### Protein isolation and Western blot analysis for MMPs and TIMPs

Total proteins from left frozen lungs of ventilated fetuses (100 mg) were isolated by sonication with cold RIPA lysis buffer containing EDTA-free protease inhibitor cocktail (Santa Cruz). Samples were centrifuged twice at 10,000 x g for 20 min at 4°C. Supernatants were retained from tissue lysates and stored at −80°C until use. Protein concentration was then measured by BCA colorimetric assay following the manufacturer’s protocol (Perbio, Erembodegem, Belgium). Equal amounts of total proteins (40 µg per lane) were reduced with LDS sample buffer containing 50 mM DTT following the manufacturer’s instructions (Life Technologies), boiled for 10 min, separated on 4–12% bis-tris polyacrylamide gels (Life Technologies) using MES SDS running buffer (Life Technologies), and transferred onto a nitrocellulose membrane (0.45 µm pore size, Bio-Rad Laboratories) by semi-dry electroblotting. Staining the membranes with 0.3% Ponceau S assessed successful transfer of proteins and molecular weight marker. After blocking with 5% nonfat powdered milk in TBS/0.1% Tween (TBS-T) for 1 hour at room temperature, membranes were incubated with the aforementioned anti-MMP2, anti-MMP14, anti-TIMP1 or anti-TIMP2 antibodies that were diluted in blocking buffer (1:1000, 1:100, 1:100, and 1:100 respectively). After overnight incubation at 4°C, membranes were washed in TBS-T and incubated with horseradish peroxidase–conjugated anti-mouse or anti-goat IgG antibody (#31430 and #31400, Perbio; 1:50,000). Antigen–antibody complexes were detected using a chemiluminescent substrate (Perbio), before exposure to X-ray film. Membranes were stripped (Restore Western Blot Stripping Buffer, Perbio), and reprobed with mouse monoclonal anti-GAPDH (G8795, Sigma-Aldrich, Diegem, Belgium; 1:5000) as an internal loading control remaining stable under the current experimental conditions. Signal intensities for MMPs and TIMPs were quantified by densitometry using the integral of the entire optical density profile (ImageJ software), and normalized to those of GAPDH. Western blot analysis was realized twice to ensure reproducibility of results.

### Statistical analysis

Data were expressed as mean ± SEM (standard error of the mean) unless indicated. For comparisons of normalized gene expression levels, values were log_10_ transformed before statistical analysis [45]. Unpaired Student’s t-test was used for all comparisons between non-operated and SHAM groups and between ventilated and unventilated lungs after ensuring normality and equality of variance. Comparisons between surgical groups (SHAM, DH, DH+ TO, TO) regarding LBWR were assessed with Kruskal-Wallis test, as the distribution was not normal. The differences between surgical groups regarding fetal body weights, histological data, protein levels and normalized gene expression levels were analyzed using one-way analysis of variance (ANOVA) after ensuring normality and equality of variance. In case of significant differences between groups, a Bonferroni post hoc correction was applied for the following meaningful comparisons: DH+ TO vs DH, DH+ TO vs SHAM, DH vs SHAM, and TO vs SHAM. The relationships between Cq values of HKGs and between expression levels of target genes were assessed by Pearson’s correlation coefficients (r), as data were normally distributed. Statistical significance was defined as *P* < 0.05. All statistical analyses were two-tailed and were performed using the SigmaStat software program (Jandel Scientific, San Rafael, CA).

## Results

### Lung and body weights

Fetal body weights did not differ between surgical groups neither between both sets of experiments (Table 2). The LBWR of SHAM fetuses was indistinguishable from that of non-operated fetuses (Table 3). The LWBR was reduced by 34% after DH creation as compared to SHAM (*P* < 0.001). In DH+ TO fetuses the LBWR increased by 227% as compared to DH fetuses (*P* < 0.001). TO performed in fetuses with intact diaphragm increased the LBWR by 205% as compared with SHAM (*P* = 0.004). The LBWR was similar between both sets of experiments (Table 3).

**Table 2:** Fetal body weights obtained from unventilated and ventilated fetuses.

**Study groups**	**All fetuses**	**Unventilated fetuses**	**Ventilated fetuses**
Non-operated	39.5 ± 2.6	38.5 ± 4.6	40.5 ± 3.5
SHAM	39.0 ± 2.7	42.7 ± 3.3	35.9 ± 3.6
DH	38.1 ± 2.3	40.1 ± 1.5	35.6 ± 4.8
DH+TO	36.1 ± 2.9	40.1 ± 3.1	35.9 ± 3.6
TO	38.9 ± 1.7	39.8 ± 3.6	38.2 ± 1.1

Values expressed as mean ± SEM. In both sets of experiments (ventilated and unventilated fetuses), 4 to 5 animals per group were included. One-way ANOVA for comparisons between surgical groups (SHAM, DH, DH+ TO, and TO) and unpaired Student’s t-test for comparisons between non-operated and SHAM fetuses and between ventilated and unventilated fetuses. Values were similar between groups and sets of experiments. SHAM, sham-operated fetuses; DH, diaphragmatic hernia fetuses; DH+ TO, DH fetuses with tracheal occlusion; TO, sham-DH fetuses with TO.

**Table 3:** Lung to body weight ratios (%) obtained from unventilated and ventilated fetuses.

**Study groups**	**All fetuses**	**Unventilated fetuses**	**Ventilated fetuses**
Non-operated	1.91 (1.85–2.02)	1.88 (1.85–2.07)	1.96 (1.84–2.01)
SHAM	1.72 (1.63–1.8)	1.64 (1.61–1.68)	1.74 (1.72–2)
DH	1.12 (1.05–1.17)^a^	1.15 (1.09–1.16)^d^	1.16 (1.06–1.27)^a^
DH+TO	2.63 (2.41–2.87)^a,b^	2.66 (2.53–2.82)^b^	2.60 (2.42–2.84)^b^
TO	3.47 (2.57–3.91)^a^	2.45 (2.24–2.79)^c^	3.91 (3.51–4.3)^a^

Values expressed as median (P25 – P75). For both sets of experiments (ventilated and unventilated fetuses), 4 to 5 animals per group were included. Kruskal-Wallis for comparisons between surgical groups (SHAM, DH, DH+ TO, and TO) followed by post hoc correction. Unpaired Student’s t-test showed no statistical differences between non-operated and SHAM fetuses neither between ventilated and unventilated fetuses. SHAM, sham-operated fetuses; DH, diaphragmatic hernia fetuses; DH+ TO, DH fetuses with tracheal occlusion; TO, sham-DH fetuses with TO. ^a^ *P* < 0.05 vs SHAM; ^b^ *P* < 0.05 vs DH; ^c^ *P* = 0.058 vs SHAM; ^d^ *P* = 0.052 vs SHAM.

### Transcriptional profiles of candidate housekeeping genes

Prior to *genorm* analysis, Cq values obtained on whole fetal lung tissue were ranged in increasing order to illustrate the different abundance classes to which candidate housekeeping genes belong. Values varied from 17 for ACTB to 26 for HPRT, which was below the cut-off value of 35, thus allowing the pursuit of the analysis in reliable conditions. When all the samples included in the study were considered, ACTB was expressed at the highest level (i.e. lowest average Cq), followed by RPLP0, ATP5B, GAPDH, and B2M. PGK1, TOP1 and SDHA showed intermediate average Cq values. HMBS and HPRT were expressed at the lowest level (i.e. highest average Cq). A similar pattern of expression was observed across the 5 experimental groups and in both sets of experiments (Figure S3) Correlations between Cq point out the potential most stable and least stable HKGs, since the best-correlated pairs are usually the most constant ones. Strong associations between Cq values were seen between SDHA and TOP1 (r = 0.83; *P* < 0.001) and between SDHA and RPLP0 (r = 0.81; *P* < 0.001). Associations between HMBS and the other HKGs were the weakest (Table S5).

### Optimal housekeeping genes for normalization

A global analysis of HKG expression stability (i.e. constancy) was initially performed with all samples included in the study. All ten HKGs exhibited low expression variability (M below 0.5) with SDHA and TOP1 identified as the most stable genes, and HMBS, ACTB, and HPRT as the least stable genes. The combination of the 2 most stable HKGs (SDHA and TOP1) had a pair-wise variation value lower than the cut off value V of 0.15. In order to assess HKG expression stability in conditions that were relevant to the current experimental protocol, different set-ups were next analyzed including all unventilated fetuses, all ventilated fetuses, non-operated and SHAM fetuses, SHAM and DH fetuses, DH and DH+ TO fetuses, SHAM and DH+ TO fetuses, and SHAM and TO fetuses. In all set-ups, the ten HKGs showed satisfying expression stability with RPLP0, ATP5B, TOP1, and SDHA usually identified in the top 4 most stable HKGs and HBMS as the most variable HKG. ACTB was the second most stable HKG in unventilated lungs whereas it was ranked as the second least stably expressed HKG in ventilated lungs. In all combinations of surgical groups, ACTB was also found in the top 4 least stable HKGs usually together with HPRT and/or PGK1. In addition to the identification of the best HKGs, the optimal number of genes necessary for normalization was assessed in all set-ups and both sets of experiments. In each case the combination of the 2 most stable HKGs was under the cut-off value V of 0.15, indicating there was no additional advantage to add a third HKG to improve the normalization factor. Results for M and V analyses are reported in the Supporting Information (Figure S4-S5; Table S6). Taking these results together, SDHA and TOP1 were used as the most appropriate set of HKGs to normalize qPCR data in the rabbit model for CDH and TO.

### Effects of lung tissue stretch on normalized levels of candidate housekeeping genes

**Figure 2:**
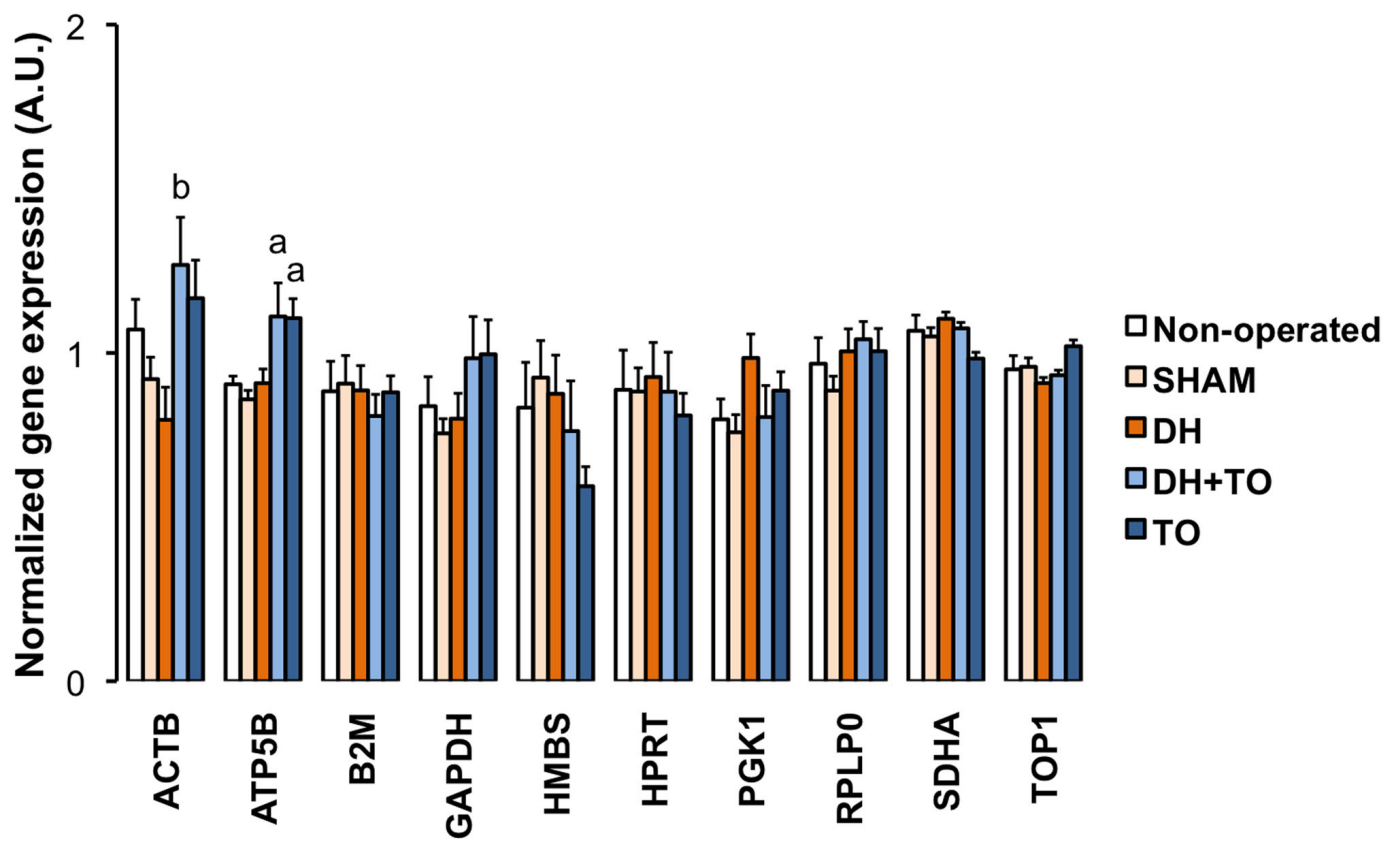
Normalized expressions of the ten candidate housekeeping genes in the fetal rabbit model.

**Figure 3:**
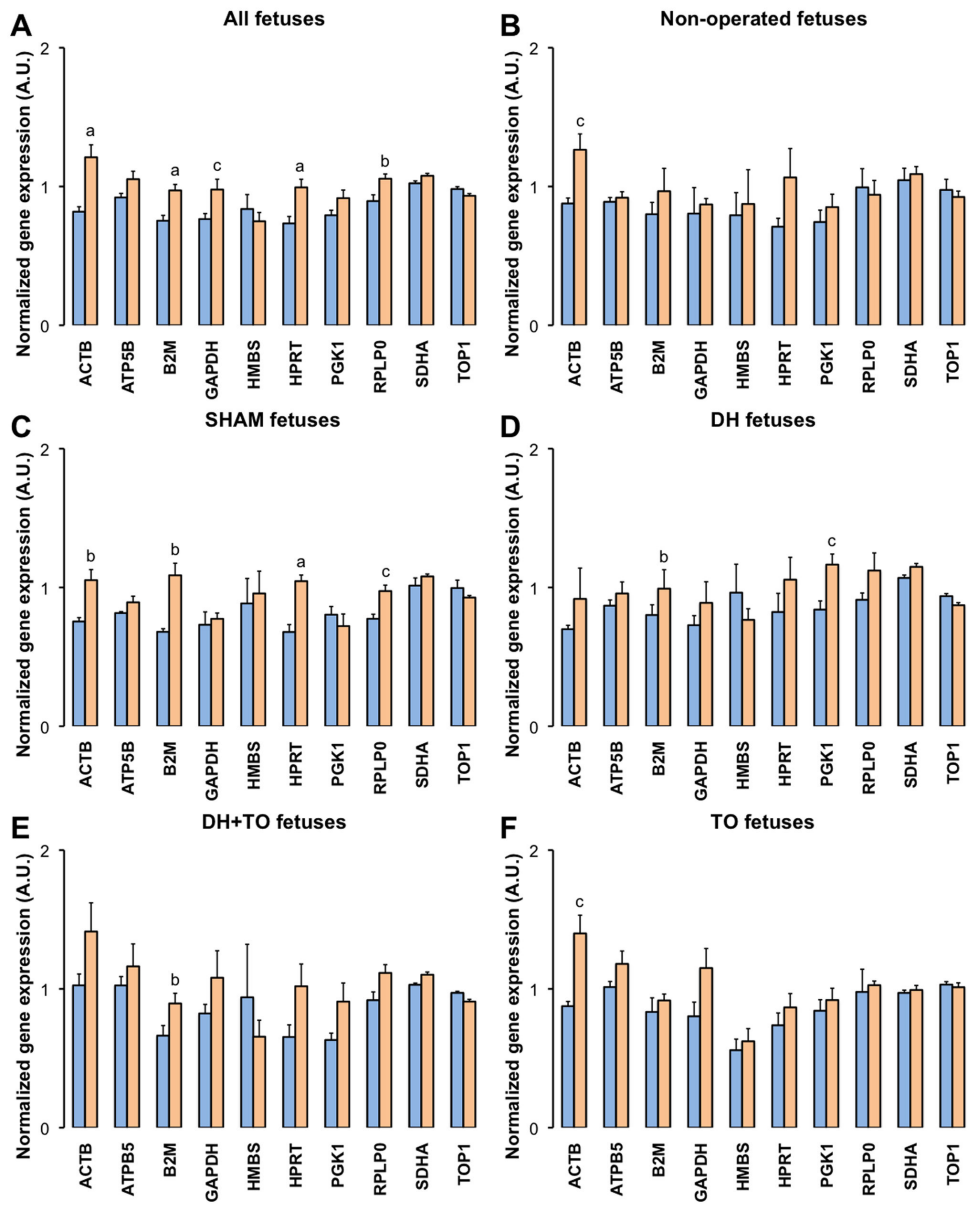
Effects of ventilation on normalized expressions of the ten candidate housekeeping genes. Mean Cq values were converted into linear values according to the efficiency corrected model, and normalized to the geometric mean of SDHA and TOP1 as calculated by *genorm*. Error bars indicate the standard error of the mean, with 4 to 5 animals per study group (blue square = unventilated lungs; orange square = ventilated lungs). **A**. Effect of ventilation in all groups combined (all unventilated lungs, *n* = 21; all ventilated lungs, *n* = 22). **B**. Effect of ventilation in non-operated fetuses. **C**. Effect of ventilation in sham-operated fetuses (SHAM). **D**. Effect of ventilation in diaphragmatic hernia fetuses (DH). **E**. Effect of ventilation in DH fetuses with tracheal occlusion (DH+ TO). **F**. Effect of ventilation in sham DH fetuses with TO (TO). Unpaired Student’s t-test was used. ^a^ *P* < 0.001; ^b^ *P* < 0.01; ^c^ *P* <0.05. A.U. = arbitrary unit.

The normalized gene expression (mRNA) levels of the ten HKGs were studied in the 5 experimental groups (Figure 2). TO upregulated mRNA levels for ACTB (*P* < 0.05 DH+ TO vs DH) and ATP5B (*P* = 0.013 DH+ TO vs SHAM; *P* = 0.012 TO vs SHAM). HKG expressions were not modulated after DH creation or sham operation. Moreover the effect of mechanical ventilation was assessed by comparisons between both sets of experiments. When all lung samples were considered (Figure 3, panel A), ventilation increased mRNA levels for ACTB (*P* < 0.001), B2M (*P* < 0.001), GAPDH (*P* = 0.014), HPRT (*P* < 0.001), and RPLP0 (*P* = 0.002). Intragroup comparisons were next performed to confirm these transcriptional effects among each surgical group (Figure 3, panels B to F). ACTB was upregulated after ventilation in non-operated (*P* = 0.014), SHAM (*P* = 0.007), and TO lungs (*P* = 0.011). Mechanical ventilation elevated mRNA levels for B2M in SHAM (*P* = 0.002), DH (*P* = 0.038), and DH+ TO lungs (*P* = 0.01). Increased mRNA levels for HPRT and RPLP0 were seen in ventilated SHAM lungs (*P* = 0.001 and *P* = 0.016 respectively). Significant upregulation for GAPDH was not further confirmed despite apparent higher mRNA levels in ventilated DH, DH+ TO, and TO lungs. On the contrary, mRNA levels for PGK1 were elevated after ventilation in DH lungs (*P* = 0.018), but this difference was found neither in other experimental groups nor in the global analysis.

Mean Cq values were converted into linear values according to the efficiency corrected model, and normalized to the geometric mean of SDHA and TOP1 as calculated by *genorm*. Error bars indicate the standard error of the mean, with 8 to 9 animals per study group. One-way ANOVA with Bonferroni correction was used for comparisons between surgical groups (SHAM, DH, DH+ TO, and TO) and unpaired Student’s t-test for comparisons between non-operated and SHAM fetuses. SHAM, sham-operated fetuses; DH, diaphragmatic hernia fetuses; DH+ TO, DH fetuses with tracheal occlusion; TO, sham DH fetuses with TO. ^a^ *P* < 0.05 vs SHAM; ^b^ *P* < 0.05 vs DH. A.U. = arbitrary unit.

### Pulmonary elastin deposition in the rabbit model for CDH undergoing TO

**Figure 4:**
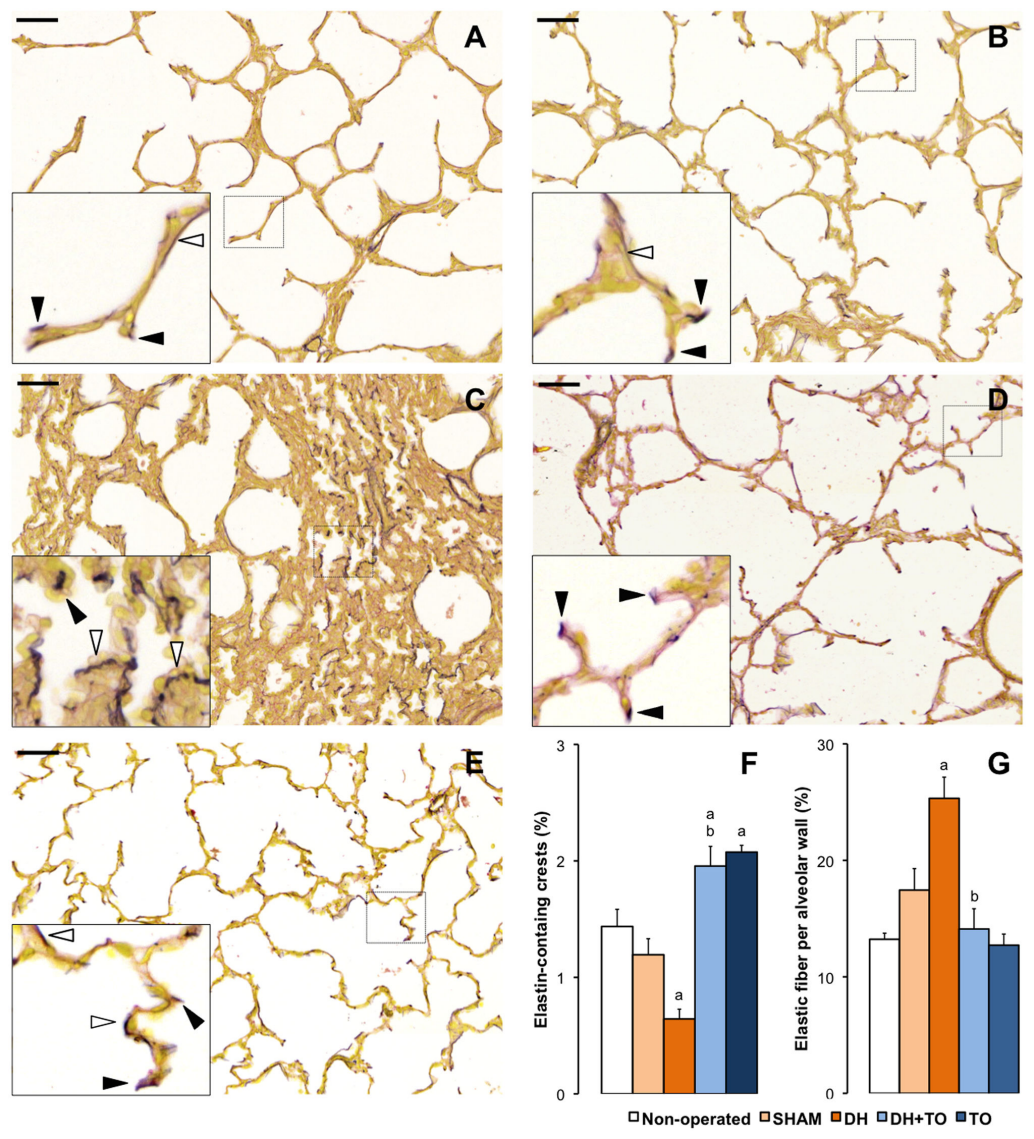
Pulmonary elastin pattern in the fetal rabbit model for CDH undergoing TO. Miller’s elastic stain colored elastic fibers in black, and smooth muscle and cytoplasm in yellow. High-magnification insets show strong accumulation of elastic foci at the tips of secondary crests in the lungs of non-operated (**A**), SHAM (**B**), DH+ TO (**D**), and TO fetuses (**E**). DH lungs showed a disorganized pattern with predominant thick elastic fibers within the alveolar walls (**C**). Solid arrowheads indicate elastin-containing secondary crests. Open arrowheads indicate elastic fibers in the alveolar walls. **F**. The mean percentage of elastin-containing crests is decreased in DH lungs and restored by TO. **G**. The mean percentage of elastic fibers in the alveolar walls was increased in DH lungs and returned to SHAM values in DH+ TO lungs. Error bars represent the standard error of the mean, with 4 to 5 animals per study group. One-way ANOVA with Bonferroni correction was used for comparisons between surgical groups (SHAM, DH, DH+ TO, and TO) and unpaired Student’s t-test for comparisons between non-operated and SHAM fetuses. SHAM, sham-operated fetuses; DH, diaphragmatic hernia fetuses; DH+ TO, DH fetuses with tracheal occlusion; TO, sham DH fetuses with TO. ^a^ *P* < 0.05 vs SHAM; ^b^ *P* < 0.05 vs DH. Scale bars = 50 µm.

During normal lung development, elastin accumulates at the apex of developing secondary septa and helps for alveolar formation [6]. The elastic fiber pattern was therefore observed in the fetal rabbit model for CDH undergoing TO (Figure 4). In non-operated lungs, elastin accumulated at the tips of secondary crests and to a lesser extent in the alveolar walls. As compared to non-operated lungs, SHAM lungs presented with a lower percentage of elastin-containing crests and a higher percentage of elastic fibers within alveolar walls, however not significant (*P* = 0.274 and *P* = 0.318 vs non-operated). As compared to SHAM lungs, elastin showed abnormal deposition in DH lungs with a reduced percentage of elastin-containing crests (*P* = 0.025) and an increased percentage of elastic fibers in the alveolar walls (*P* = 0.043), which looked thickened. In DH+ TO lungs, the relative abundance of elastin-containing secondary crests was increased as compared to DH lungs (*P* = 0.01), above the level of SHAM lungs (*P* = 0.017). Moreover TO decreased the percentage of elastic fibers in the alveolar walls of DH lungs (*P* = 0.004) to a comparable level to that of the SHAM lungs. Finally SHAM lungs subjected to TO showed an increased in the relative abundance of elastin-secondary crests (*P* = 0.013). TO also tended to reduce the percentage of elastic fiber within the alveolar walls, but differences with SHAM lungs were not significant (*P* = 0.161).

### Alveolarization genes in the rabbit model for CDH undergoing TO

**Figure 5:**
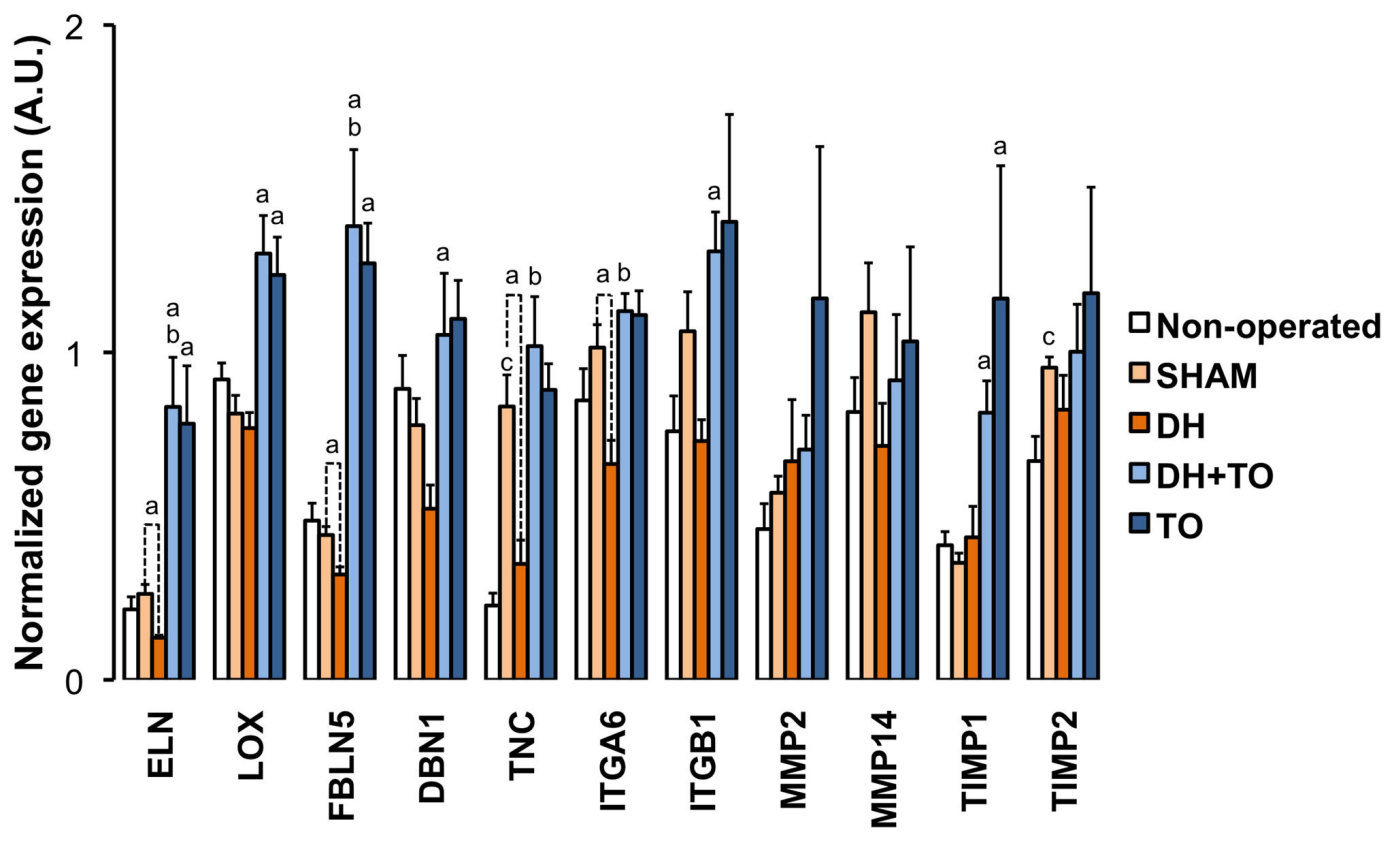
Normalized expression of eleven alveolarization genes in the fetal rabbit model for CDH undergoing TO. Mean Cq values were converted into linear values according to the efficiency corrected model, and normalized to the geometric mean of SDHA and TOP1 as calculated by *genorm*. Error bars illustrate the standard error of the mean, with 4 to 5 animals per study group. One-way ANOVA with Bonferroni correction was used for comparisons between surgical groups (SHAM, DH, DH+ TO, and TO) and unpaired Student’s t-test for comparisons between non-operated and SHAM fetuses. SHAM, sham-operated fetuses; DH, diaphragmatic hernia fetuses; DH+ TO, DH fetuses with tracheal occlusion; TO, sham DH fetuses with TO. ^a^ *P* < 0.05 vs SHAM; ^b^ *P* < 0.05 vs DH; ^c^ *P* < 0.05 vs non-operated. A.U. = arbitrary unit.

Expression levels of target genes regulating alveolar formation were assessed by qPCR in the fetal rabbit model for CDH undergoing TO, using the geometric mean of SDHA and TOP1 for normalization of qPCR data (Figure 5). Gene expression analyses focused on specific ECM-related genes involved in the normal process of alveolar formation, including genes encoding matrix components peaking during alveolarization (ELN, TNC) [46,47]; genes regulating the assembly and stability of various ECM molecules like elastin fibers (LOX, FBLN5) [48]; genes controlling rearrangements of the actin-cytoskeleton to modulate cell migration through the surrounding ECM (ITGA6, ITGB1 and DBN1) [49–51]; and genes encoding MMPs and TIMPs that are involved in the thinning of pulmonary interstitium and the formation of a functional blood/gas barrier [52]. Special consideration was given to MMP2 and MMMP14 due to their crucial role during the alveolar stage [42,53–55]. MMP9 was not assessed, as its activity remains stable during the alveolarization process in the fetal rabbit lung [42]. TIMP2 was chosen as it is involved in the regulation of MMP2 activation together with MMP14, whereas TIMP1 was assessed as the prototypic inhibitor of most MMPs.

**Figure 6:**
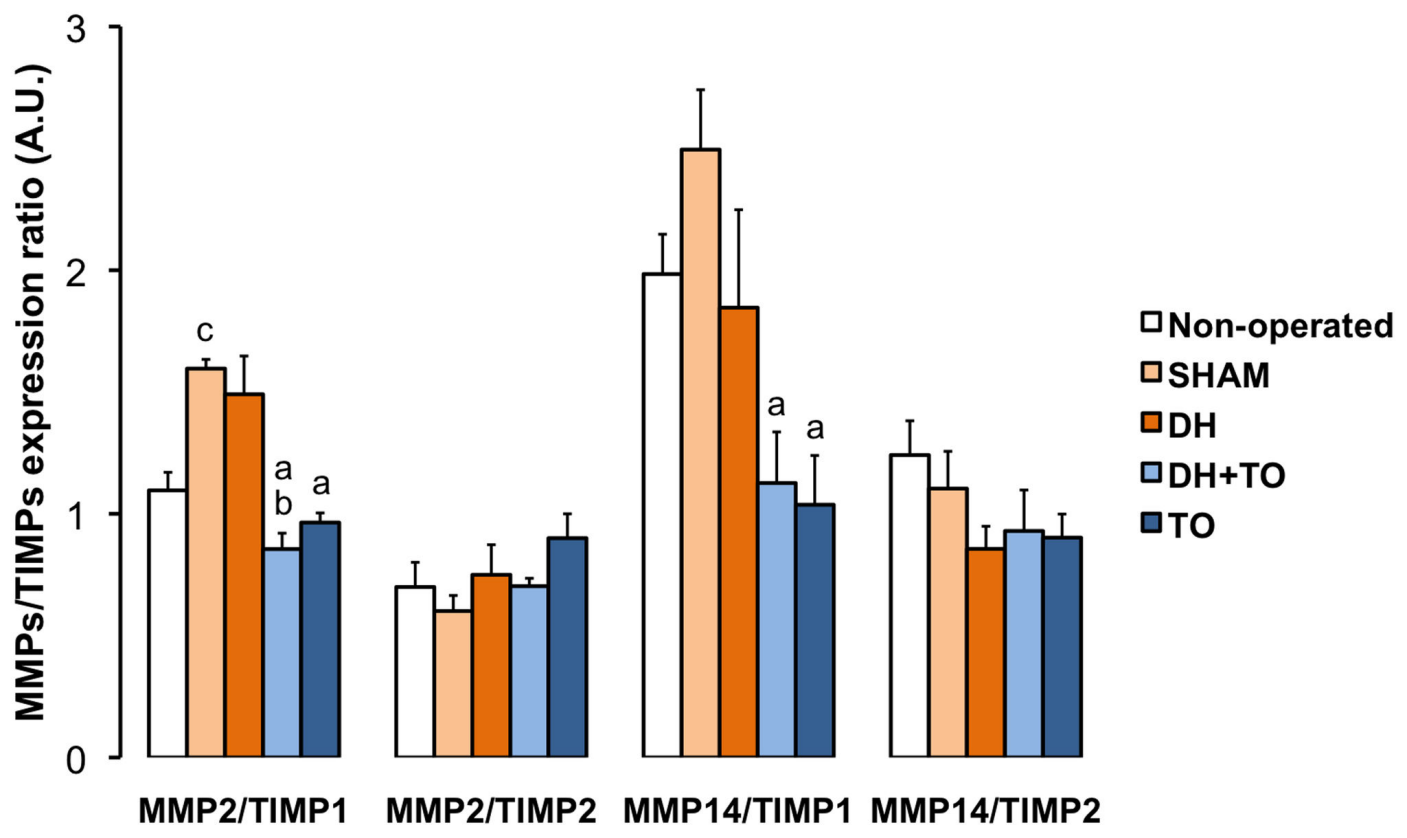
Gene expression ratios between MMPs and TIMPs in the fetal rabbit model for CDH undergoing TO. Mean Cq values for each gene were converted into linear values according to the efficiency corrected model, and normalized to the geometric mean of SDHA and TOP1 as calculated by *genorm*. Normalized values for MMP2 and MMP14 were then expressed relative to normalized values for TIMP1 and TIMP2. Error bars illustrate the standard error of the mean, with 4 to 5 animals per study group. One-way ANOVA with Bonferroni correction was used for comparisons between surgical groups (SHAM, DH, DH+ TO, and TO) and unpaired Student’s t-test for comparisons between non-operated and SHAM fetuses. SHAM, sham-operated fetuses; DH, diaphragmatic hernia fetuses; DH+ TO, DH fetuses with tracheal occlusion; TO, sham DH fetuses with TO. ^a^ *P* < 0.05 vs SHAM; ^b^ *P* < 0.05 vs DH; ^c^ *P* < 0.05 vs non-operated. A.U. = arbitrary unit.

In comparison to SHAM lungs, DH lungs showed significant reduced mRNA levels for ELN (*P* < 0.001), FBLN5 (*P* = 0.032), TNC (*P* = 0.011), and ITGA6 (*P* = 0.008). DBN1 and ITGB1 mRNA levels tended to decrease in DH lungs (by 33% and 31% respectively), but differences with SHAM lungs were not longer significant after Bonferroni correction. In comparison with DH lungs, DH+ TO lungs exhibited restored mRNA levels for ELN (*P* < 0.001), FBLN5 (*P* < 0.001), TNC (*P* = 0.009), and ITGA6 (*P* = 0.001), which were above the level of SHAM lungs for ELN and FBLN5 (*P* = 0.001 and *P* < 0.001, respectively). In addition mRNA levels for genes that were not significantly modulated in DH lungs were increased in the DH+ TO lungs as compared with SHAM lungs, including LOX (*P* = 0.002), DBN1 (*P* = 0.019), ITGB1 (*P* = 0.027), and TIMP1 (*P* = 0.008). In comparisons to the SHAM group, ELN, LOX, FBLN5, and TIMP1 were overexpressed in the TO group (*P* = 0.003, *P* = 0.011, *P* < 0.001, and *P* = 0.009, respectively). Gene expression levels for MMP2, MMP14 and TIMP2 were similar between the surgical groups. Ratios between MMPs and TIMPs were then assessed to appraise ECM homeostasis. Because all TIMPs may theoretically inhibit all MMPs, all ratios were considered. Besides the classically described combinations between MMP2 and TIMP2 and between MMP9 and TIMP1, it has been shown that TIMP1 may also bind MMP2 [56]. The calculated ratios between MMP2/TIMP1 mRNA levels (Figure 6) were significantly decreased in DH+ TO lungs (*P* = 0.001 vs DH; *P* < 0.001 vs SHAM) and TO lungs (*P* = 0.003 vs SHAM). The MMP14/TIMP1 ratio was also reduced in DH+ TO and TO lungs as compared with SHAM lungs (*P* = 0.01 and *P* = 0.009 respectively). In comparison with non-operated lungs, SHAM lungs presented with overexpressions for TNC (*P* = 0.004) and TIMP2 (*P* = 0.026), and higher MMP2/TIMP1 ratio (*P* = 0.001). The LBWR was positively correlated with FBLN5 (r = 0.71, *P* < 0.001), DBN1 (r = 0.65, *P* < 0.001), LOX (r = 0.61, *P* = 0.002), TNC (r = 0.58, *P* = 0.011), to a lesser extent, with ITGA6 (r = 0.46, *P* < 0.001) and ELN (r = 0.44, *P* = 0.032). The LBWR was negatively correlated with MMP2/TIMP1 ratio (r = -0.56, *P* = 0.006). Genes with strong correlated mRNA levels are often regulated by a common mechanism [57]. Correlations were therefore established to highlight potential functional links between target genes (Table 4) [57]. ITGB1 was positively correlated with all the other genes excepted for DBN1. Only ITGB1 was correlated with MMPs and TIMPs. Positive associations were also seen between MMPs and TIMPs. Strong to moderate correlations were observed between ELN, LOX, FBLN5, DBN1, TNC, and ITGA6. No relationship was established between mRNA levels of elastogenesis-related genes and those of MMPs and TIMPs.

**Table 4:** Bivariate Pearson’s correlation coefficients between the eleven target genes based on normalized mRNA levels obtained for all ventilated samples (*n* = 22).

Gene	ELN	LOX	FBLN5	TNC	DBN1	ITGA6	ITGB1	MMP2	MMP14	TIMP1	TIMP2
ELN											
LOX	0.50^c^										
FBLN5	0.49^c^	0.90^a^									
TNC	0.59^c^	0.42	0.55^c^								
DBN1	0.22	0.58^b^	0.74^a^	0.40							
ITGA6	0.47^c^	0.65^a^	0.67^a^	0.59^b^	0.64^a^						
ITGB1	0.61^b^	0.57^b^	0.50	0.51^c^	0.20	0.70^a^					
MMP2	0.23	-0.04	-0.07	0.02	-0.06	0.27	0.59^b^				
MMP14	0.17	-0.03	-0.19	0.28	0.02	0.20	0.43^c^	0.49^c^			
TIMP1	0.36	0.20	0.14	0.15	0.10	0.41	0.69^a^	0.85^a^	0.38		
TIMP2	0.34	0.08	-0.04	0.37	-0.19	0.31	0.76^a^	0.84^a^	0.62^b^	0.75^a^	

^a^ *P* < 0.001; ^b^ *P* < 0.01; ^c^ *P* < 0.05.

### Effect of different normalization strategies on target gene expression

To illustrate the consequences of suboptimal HKG selection, expression levels of the aforementioned target genes were normalized using single HKGs, including the most variable gene (HMBS), a gene overexpressed by TO (ACTB), a gene with an intermediate variability of expression (ATP5B), and the 2 most constant genes (TOP1 and SDHA). As shown in Figure S6, the choice of improper HKG affected dramatically statistical significance. ACTB reduced the impact of TO so much so that differences between groups could not be evidenced anymore. The use of HMBS had globally similar consequences. Moreover overexpression of MMP2 after TO was observed with HMBS, which was absent in the case of proper normalization with both SDHA and TOP1 as suggested by the *genorm* software. ATP5B also failed to show significant differences between groups for some of the tested target genes. Finally the use of SDHA or TOP1 as single normalizers led to similar changes –yet not identical-as compared by the normalization by both SDHA and TOP1. However, some statistical differences could not be obtained anymore mainly with TOP1 and to a lesser extent with SDHA.

### Localization and abundance of expression of MMPs and TIMPs in the rabbit model for CDH undergoing TO

**Figure 7:**
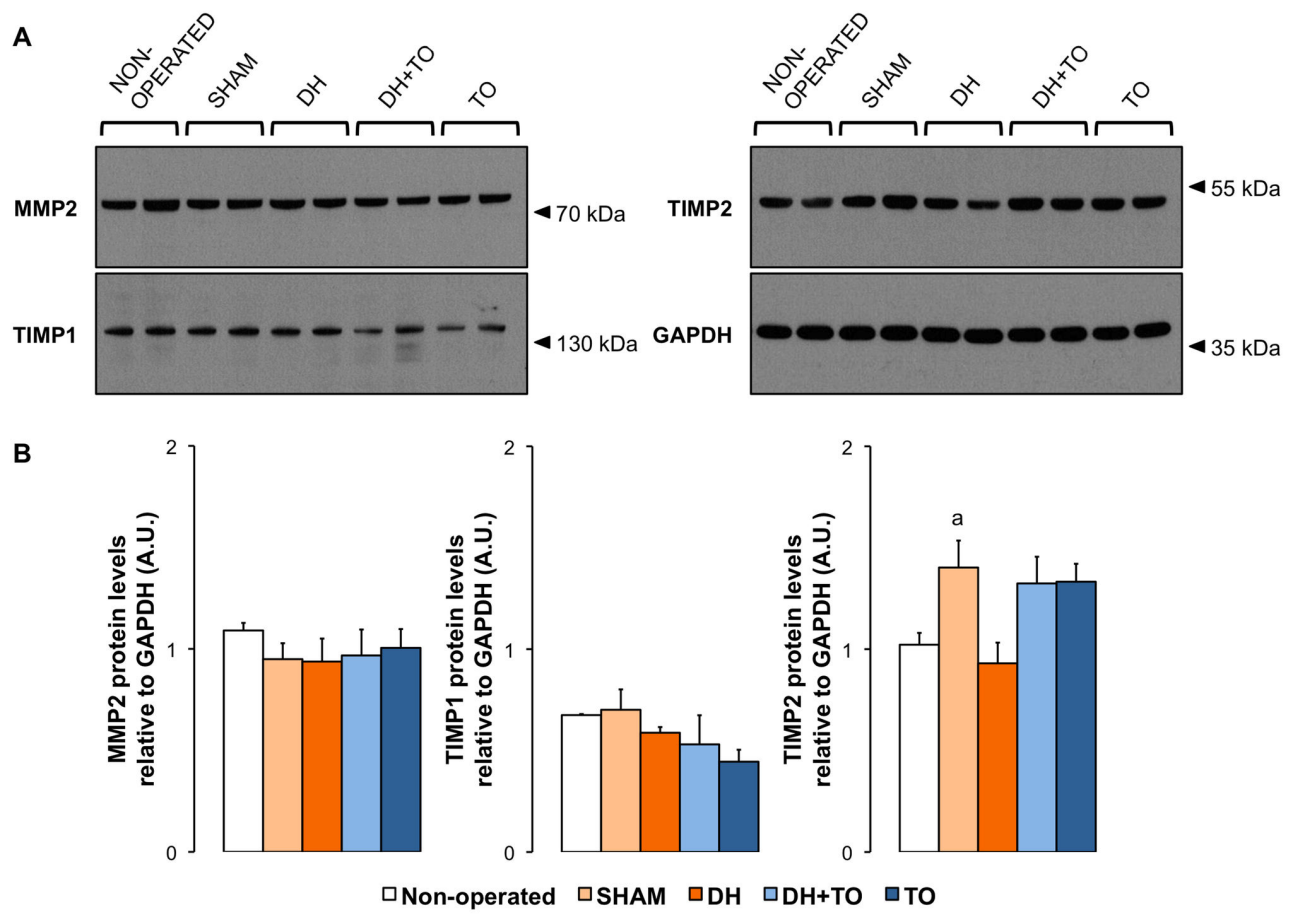
Protein expression levels of MMPs and TIMPs in lung tissue homogenates in the fetal rabbit model for CDH undergoing TO. **A**. Representative Western blot detection for MMP2, TIMP1, and TIMP2 in non-operated, SHAM, DH, DH+ TO, and TO groups. GAPDH was used as loading control. Western blot analyses revealed the latent form of MMP2 (72 kDa) and high-molecular weight complexes for TIMP1 (130 kDa) and TIMP2 (54 kDa). Two representative samples for each group are depicted. **B**. Densitometric analysis showing that TIMP2 dimeric complex was upregulated in the lung of SHAM fetuses as compared to non-operated fetuses. Values and error bars illustrate mean and standard error of the mean obtained from non-operated (*n* = 4), SHAM (*n* = 4), DH (*n* = 3), DH+ TO (*n* = 4), and TO groups (*n* = 4). One-way ANOVA with Bonferroni correction was used for comparisons between surgical groups (SHAM, DH, DH+ TO, and TO) and unpaired Student’s t-test for comparisons between non-operated and SHAM fetuses. SHAM, sham-operated fetuses; DH, diaphragmatic hernia fetuses; DH+ TO, DH fetuses with tracheal occlusion; TO, sham DH fetuses with TO. ^a^ *P* < 0.05 vs non-operated. A.U. = arbitrary unit.

Besides transcriptional regulation MMPs and TIMPs may be controlled at translational and post-translational levels. Firstly, protein levels for MMP2, MMP14, TIMP1, and TIMP2 in lung tissue samples homogenated with EDTA-free buffer were evaluated by Western blot under reducing and denaturing conditions to determine whether differences in gene expression translated into differences in protein expression in the fetal rabbit model for CDH undergoing TO (Figure 7). In all study groups, immunoreactive bands for MMP2 were seen at 72 kDa corresponding to its latent form. Despite the ability of the antibody to recognize the active form of MMP2 [42], the cleaved protein was not evidenced. Consistent with mRNA levels, densitometric analysis showed no differences in pro-MMP2 levels between study groups. For MMP14 a single band was visualized at 22 kDa, compatible with a shedding fragment. Because this band appeared in all Western blot experiments and all groups, it is less likely to represent an artifact. However the expression of this fragment varied noticeably within each group preventing from any robust conclusion in the current experimental model (Figure S7). Regarding protein levels of TIMPs, western blot yielded high-molecular-weight species at 130 kDa for TIMP1 and 54 kDa for TIMP2, which could arise from MMP/TIMP1 complexes and TIMP2 dimers, respectively. Free forms of TIMPs were not detected. Densitometric analysis for MMP2, TIMP1, and TIMP2 revealed a significant increase in the dimeric complex of TIMP2 in SHAM lungs by 37% as compared to non-operated lungs (*P* = 0.033).

**Figure 8:**
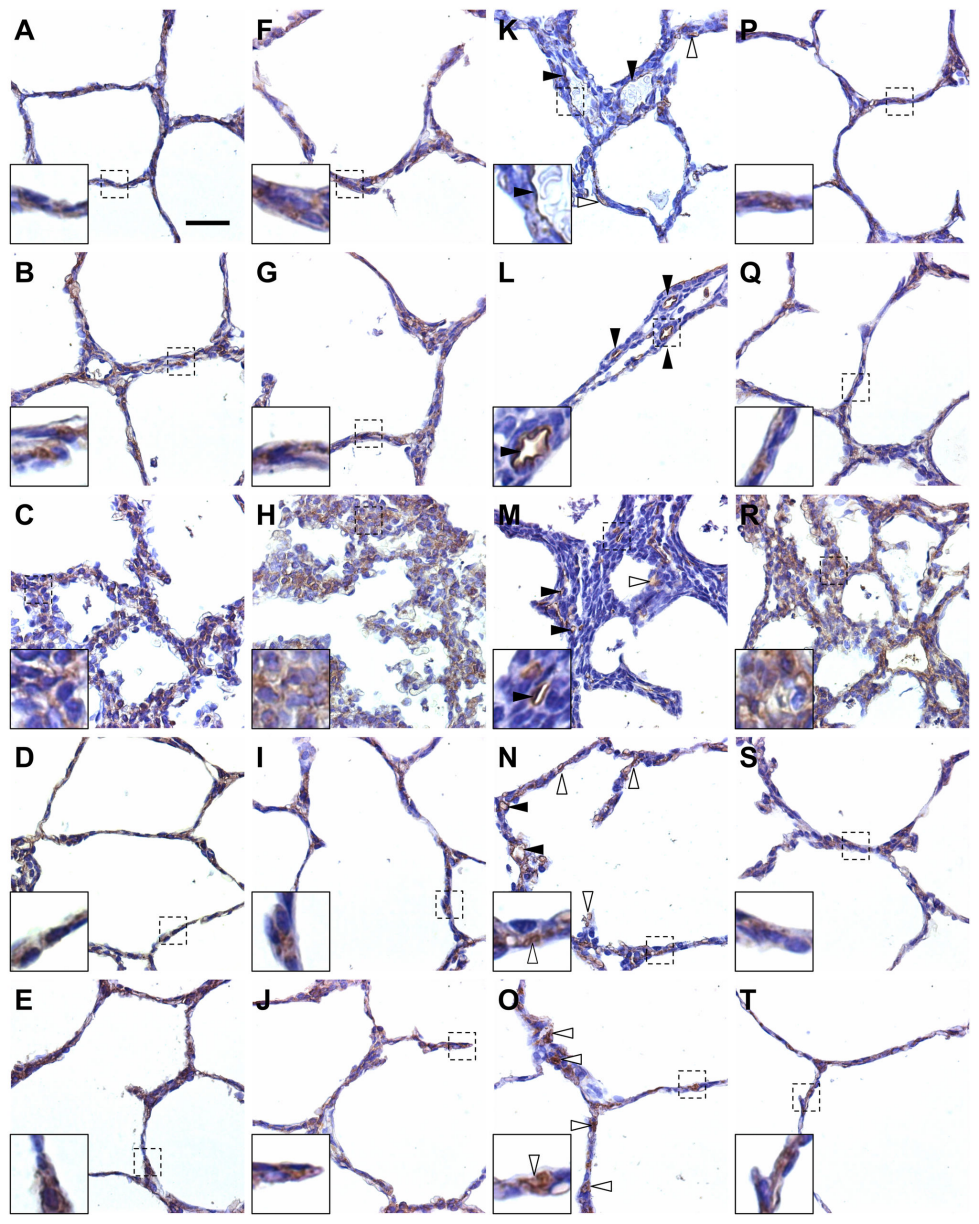
Localization of MMPs and TIMPs in alveolar septa in the fetal rabbit model for CDH undergoing TO. Representative images of immunohistochemistry for MMP2 (**A**–**E**), MMP14 (**F**–**J**), TIMP1 (**K**–**O**), and TIMP2 (**P**–**T**) in non-operated (**A**, **F**, **Q**, **P**), SHAM (**B**, **G**, **L**, **K**), DH (**C**, **H**, **M**, **R**), DH+ TO (**D**, **I**, **N**, **S**), and TO lungs (**E**, **J**, **O**, **T**). MMP2, MMP14, and TIMP2 were expressed in alveolar epithelial cells in non-operated, SHAM, DH+ TO, and TO lungs (high-magnification inserts). MMP2, MMP14, and TIMP2 were also shown in mesenchymal cells in the thickened septa of DH lungs (diffuse staining in-high magnification inserts). TIMP1 was localized in endothelial cells of capillaries in all study groups (solid arrowheads), and in some alveolar epithelial cells (open arrowheads) in non-operated, SHAM, and DH lungs (**K**, **L**, **M**). DH+ TO (**N**) and TO lungs (**O**) showed a much more diffuse immune reaction for TIMP1 in alveolar epithelial cells (open arrowheads) as compared to non-operated and SHAM lungs. Scale bar = 50 µm in A through T.

Secondly, cellular localization of MMP2, MMP14, TIMP1, and TIMP2 was assessed by immunohistochemistry to better illustrate their roles regarding alveolar development in the fetal rabbit model for CDH undergoing TO (Figure 8). A co-localization of MMP2, MMP14 and TIMP2 was expected in the peripheral parenchyma, as the activation of MMP2 requires MMP14 and TIMP2. Indeed MMP2, MMP14, and TIMP2 were diffusely co-expressed in alveolar epithelial cells of non-operated, SHAM, DH+ TO, and TO lungs. In DH lungs, their immunoreactivity was also observed in mesenchymal cells of thickened septa. Only TIMP1 showed a different pattern of distribution after TO. In non-operated and SHAM lungs, TIMP1 immunoreactivity was found in basement membranes underlying the alveolar epithelium, endothelial cells of alveolar capillaries, and to a lesser extent in the cytoplasm of some alveolar epithelial cells. In hypoplastic lungs the immunoreactivity pattern of TIMP1 in endothelial and epithelial cells was comparable to that of non-operated and SHAM lungs, without detection in the interstitium of thickened alveolar septa. Strikingly alveolar walls of TO-exposed lungs exhibited a much more diffuse immune reaction for TIMP1 as compared to non-operated and SHAM lungs. Finally, the immunolocalization of MMP2, MMP14, TIMP1 and TIMP2 was similar in lung airways and vasculature in all study groups (Figure S8).

## Discussion

The main objective of this work was to assess the mechanoregulation of gene expression after TO as one of the principal mechanisms by which TO improves lung alveolarization. In the fetal rabbit model for CDH, TO stimulated the alveolar formation, notably through an increased number of secondary crests in both intact and hypoplastic lungs together with increased transcription of several genes related to the ECM. Those genes were involved in the normal process of alveolarization, including molecules expressed in the growing secondary septa or involved in interactions between cells and extracellular environment. While TO increased the expression of genes that were downregulated in DH rabbit lungs, TO also induced changes in the expression of other genes that were not affected in lung hypoplasia. Most of the transcriptional effects observed might be beneficial for alveolar formation. However, the meaning of higher levels for TIMP1 has to be further explored, as these changes might be detrimental for postnatal alveolar development.

The transcription of pulmonary genes in the present study was evaluated by qPCR as a more sensitive, reliable, and accurate tool than previously used techniques, like hybridization methods [16] and conventional end-point PCR followed by gel electrophoresis [12,15]. Accurate interpretation of qPCR results requires an appropriate methodology to minimize technical variations and highlight true biological variations between experimental groups. Each step of qPCR should therefore be controlled with careful care for sample preparation, RNA extraction, cDNA synthesis, primer design, assay validation, and normalization strategy [37]. The current gold standard for normalization is the use of stable HKGs as internal controls. Since a perfect and universal HKG does not exist, normalization by the geometric average of multiple and validated HKGs has been strongly recommended [37–40]. MIQE guidelines notwithstanding, most recent studies focusing on mRNA expression after CDH and/or TO in rodents or sheep reported normalization against a single HKG, like 18S rRNA [6], ACTB [58] or GAPDH [9]. The present study confirmed that the use of inadequate HKGs or inappropriate number of HKGs might considerably affect statistical significances. Despite apparent low HKG expression variability obtained after *genorm* analysis, mechanical stretch induced by TO or mechanical ventilation changed the transcription of some candidate HKGs. ACTB, which has been identified as an unstable HKG for human lung epithelial cells and whole lungs exposed to cyclic mechanical strain [59], was upregulated after mechanical ventilation and TO. Mechanical stretch also affected transcripts involved in energy production pathways like GAPDH and ATP5B. The metabolic effects of TO have already been suggested in intact murine lungs in which six of the key glycolytic enzyme genes were upregulated as early as one hour after TO [14]. Moreover mechanical ventilation increased B2M levels, which have been related to chronic lung disease in ventilated premature infants [60]. These results collectively indicate that special caution should be paid to the selection and validation of HKGs for qPCR studies in general, and in particular for those focusing on TO-mediated mechanotransduction. Besides the use of validated HKGs as a methodological requisite for qPCR, the identification of changes in HKG expression might also be helpful to highlight modifications of basic cellular mechanisms under specific physiopathological conditions [14], even if those cellular pathways were presumably constitutively expressed.

Besides the selection and validation of HKGs, the choice of an appropriate control group is also crucial for interpretation of results and may be the source of discrepancies in the reported literature. In the nitrofen model in fetal rats, fetuses having received olive oil were more often chosen as controls [6,10,11] than those exposed to nitrofen and free of diaphragmatic hernia. In the surgical model in fetal sheep, control fetuses were non-operated [2,6,12] or sham-operated fetuses [3]. In the surgical model in fetal rabbits, non-operated fetuses were considered as suitable controls [19,28], because non-operated and sham-operated fetuses shared similar fetal growth and pulmonary morphology [27]. However recent studies showed altered lung mechanics in sham DH rabbit fetuses [32] and lower LBWR in sham TO rat fetuses [15], raising the question of the suitability of non-operated fetuses as controls for surgical models of CDH and TO. In the present work, SHAM fetuses (i.e. undergoing two consecutive sham operations) were a priori considered as the most suitable controls for comparisons with other surgical groups because of pain, stress, thoracic scar, and possible oligoamnios induced by sham surgery. SHAM fetuses showed neither real pulmonary hypoplasia nor significant changes in elastin deposition, but sensitive qPCR could detect differences for TNC, TIMP2, and MMP2/TIMP1 ratio. Apart from pain and stress, sham surgery might also induce inflammation since increased TNC mRNA levels and imbalance between MMPs and TIMPs have been linked to inflammation in pulmonary fibrosis [61]. The importance of the choice of an appropriate control group may be illustrated by discordances regarding TNC expression between the present study and a previous work in which our team compared DH rabbit lungs (without sham TO) to non-operated fetuses [19]. Actually this contradiction may be simply explained by significant differences in TNC expression between non-operated and SHAM lungs. Taking these results into account, non-operated fetuses should not be considered anymore as an appropriate control group for studies in the surgical rabbit for CDH undergoing TO.

The present study suggests that variable increase in mRNA levels for ELN, LOX, FBLN5, DBN1, TNC and ITGA6 could lead to variable increase in pulmonary growth, indicating that lung growth induced by TO is closely related to the expression of specific alveolarization-relevant genes. Essential for secondary septation and alveolarization, elastogenesis occurs in septal myofibroblasts and results from highly regulated steps with coordinated gene expressions, which involve synthesis of the monomeric protein, cross-linking into insoluble protein, and aggregation with microfibrils [46,48]. In agreement with previous reports in ovine and murine models [6,24–26], TO increased ELN transcripts and improved the pulmonary deposition of elastin fibers in the fetal rabbit model. In addition to these confirmatory findings our new results suggest that TO might contribute to form the complete functional elastic fiber under pretranslational actions on LOX and FBLN5, both genes that were downregulated in the CDH rabbit lungs [19]. Besides elastin, myofibroblasts also produce TNC, an ECM glycoprotein that accumulates at sites of branching during lung morphogenesis and strictly collocates with elastin at the apex of secondary crests [47]. Knockout models have already shown the role of TNC in lung morphogenesis [17,62]. In addition TNC has been considered as a factor transducing mechanical forces and controlling cell migration during early lung development [62]. The CDH model in the fetal rabbit showed decreased pulmonary transcripts for TNC in concordance with previous data in the nitrofen model [63]. The restoration of TNC levels after TO and experimental data in neonatal chronic lung disease [35] support the role of TNC in alveolarization and transduction of mechanical stimuli. Apart from molecules secreted in the ECM, integrins are a wide family of transmembrane heterodimeric glycoproteins that link the cytosqueleton to the extracellular environment and act as adhesion receptors, signaling receptors, and mechanoreceptors to control cell growth, migration, and differentiation [17]. Recent descriptive studies on lung ontogeny have supported the role of integrin subunits ITGA6 and ITGB1 in the alveolarization process [49,50]. On the one hand, mice deficient for ITGA6 develop severe lung hypoplasia [17]. On the other hand, ITGB1 is highly expressed in alveolar myofibroblasts and bind FBLN5 and microfibrils during the extracellular step of elastic fiber assembly, a process occurring in close association with the cell surface [48]. The present work also suggests the involvement of ITGA6 deficiency in CDH, as TO restored ITGA6 mRNA levels concomitantly with the correction of lung hypoplasia. Moreover increased transcripts for ITGB1 after experimental TO fell in line with the overexpression of elastogenesis-related genes induced by a sustained mechanical stretch. Interestingly ITGB1 transcripts were strongly associated with MMPS and TIMPs transcripts unlike the other alveolarization genes. Previous reports have reported functional links between integrins and the MMP/TIMP system [9]. For instance ITGB1 may complex to MMPs and TIMPs, establishing a link between pericellular matrix remodeling and cytoskeletal reorganization to promote cell migration [64]. The regulation of MMP2 and TIMP1 transcription by other integrins has also been proposed [65,66], but the exact meaning of the relationships between ITGB1 and TIMPs remains to be determined in the settings of CDH and TO. Besides integrins the reorganization of the actin-cytoskeleton may be regulated by DBN1, an actin-binding protein transiently expressed by myofibroblasts at the tips of secondary septa like ELN and TNC [51]. It has been suggested that DBN1 regulates the organization of actin filaments in septal myofibroblasts, leading to the formation of cellular protrusions and finally the elongation of the secondary septum [51]. Increased DBN1 transcripts after experimental TO reinforce the idea that DBN1 participates in alveolarization.

Sparse and controversial data based on experimental models have involved the MMP/TIMP system in the defective alveolar and vascular development of CDH lungs [63,67]. In the rabbit model lung hypoplasia did not come along with changes in MMPs or TIMPs transcription. These results together with immunohistochemical data were not in favor of a ruptured MMP/TIMP balance. However physical forces may regulate the transcription of MMPs and TIMPs [9]. With regards to previous reports different types of mechanical stretch did not modify gene expression in the same manner [9]. Indeed intermittent cyclical mechanical strain imitating fetal breathing movements did not change the transcriptional levels of most MMPs or TIMPs [68], while mechanical stretch imitating injury induced MMP2, MMP14, and TIMP1 transcripts [69]. A sustained mechanical stretch after TO in the fetal rabbit model did not change mRNA or protein levels for MMPs nor their cellular localization. Surprisingly TO induced TIMP1 transcripts in whole lung tissue and increased TIMP1 immunoreactivity in alveolar septa. This seems to contradict the idea that TO might be beneficial for the remodeling of newly formed alveoli, as suggested by the well-established relationship between increased TIMP1 levels and deposition of ECM components in fibrotic pulmonary diseases [61]. Moreover potential detrimental effects of TO have been reported in the fetal lamb model for CDH, in which hypoplastic lungs treated by either sustained or temporary TO still display persistent increase in alveolar wall thickness as compared to sham-operated lungs [70]. Nevertheless a transient increase in TIMP1 mRNA levels during septation might prevent from excessive degradation of newly formed elastin [52]. Besides the inhibition of MMPs activity, TIMP1 may also induce proliferation and reduce apoptosis in different cell lines through pathways that are independent of MMP inhibition [71]. Hence overexpression of TIMP1 might be coherent with enhanced tissue proliferation after TO. These results represent a first step towards a better knowledge of the molecular events initiated by TO. However additional experiments based on enzymatic activities including the study of other MMPs and TIMPs are warranted to precise the role of the MMP/TIMP balance in the settings of CDH and TO.

**Figure 9:**
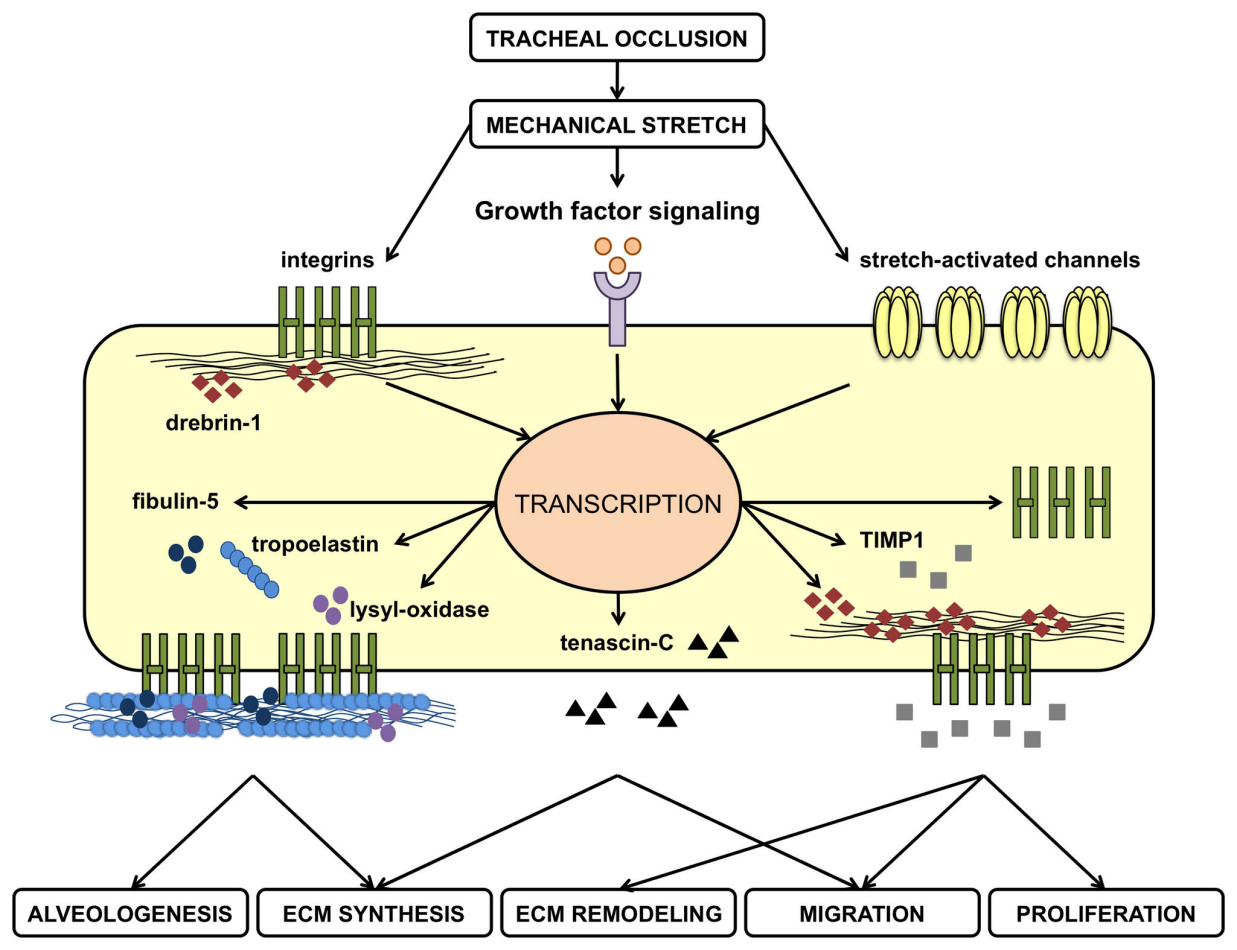
Synopsis of hypothetical mechanisms by which alveolarization-relevant genes related to the extracellular matrix (ECM) might be modulated by a sustained mechanical stretch induced by tracheal occlusion. Tissue stretch is transmitted to alveolar myofibroblasts directly by mechanoreceptors (integrins) and ion channels or indirectly by liberation of growth factors stored/secreted in the ECM. Subsequent activation of intracellular signalings leads to increased transcription of target genes linked to the ECM and involved in elastogenesis (tropoelastin, lysyl-oxidase, fibulin-5, integrins), cytoskeletal rearrangements and cell migration (integrins, drebrin1, tenascin-C), ECM remodeling (tissue inhibitor of matrix metalloproteinase-1, TIMP1) or cell proliferation (TIMP1).

We may only hypothesize about the mechanisms by which TO modulated target genes. Changes in gene expressions may occur after direct activation of a mechano-responsive gene promoter region, after activation of membrane receptors and initiation of intracellular pathways, or after liberation of growth factors in the extracellular environment with consequential paracrine or autocrine actions [8,9]. It is therefore plausible that TO might upregulates genes expression after release of growth factors [6,10–12], activation of receptors (e.g. integrins), or stimulation of mechano-sensitive ion channels, with subsequent stimulation of target genes by various transcription factors [13]. The activation of growth factor signalings after TO might explain coordinated expressions of genes that are co-regulated in favor of alveolar formation, as suggested by positive relationships between target genes observed in the present study. For instance alveolar formation requires the fibroblast growth factor-18 (FGF18), which is upregulated by TO [6] and induces ELN, LOX and FBLN5 transcripts in fetal lung fibroblasts [72]. Thus FGF18 might be a common denominator of functionally related genes encoding ELN, LOX and FBLN5. Additionally other growth factors might be involved in the relationships between matrix-related genes. After induction of a mechanical stress numerous ECM components are indeed targeted by the transforming growth factor-β (TGFβ) signaling, including ELN, TNC, TIMPs, and integrins [9]. Since TGFß-2 is increased after TO [73], activation of the TGFβ signaling could account for coordinated increased mRNA levels for TNC, ELN and ITGA6 or for ITGB1 and TIMPs. The findings of the current study as well as the proposed putative mechanisms of TO-induced alveolar formation have been summarized in Figure 9. In this hypothetical synopsis the mechanical stretch induced by TO upregulates ELN transcription together with LOX and FBLN5 in alveolar myofibroblasts for proper coordination of elastin assembly. This process is encouraged by integrins that bind microfibrils to promote mature elastic fiber formation. Besides elastic fibers new primary septa also contains TNC. Further elongation of the primary septum is stimulated by myofibroblast migration through the extracellular environment with the help of integrins, DBN1, and TNC. Additional elevated TIMP1 levels prevent from excessive degradation of newly synthesized elastin and participate to induced cell proliferation after TO. Finally we may speculate that overexpression of factors transducing mechanical forces such as integrins, TNC or DBN1 might create a positive feedback loop to increase cell sensitivity to mechanical strain.

Some limitations to this work should be acknowledged. Firstly, sample size could have been underestimated in the absence of gene expression studies in the fetal rabbit model undergoing TO. Indeed, additional numbers of subjects would have probably affected the results significantly for a few comparisons with statistical power between 72 and 84% (e.g. for DBN1, ITGB1, and MMP14/TIMP1 ratio). Yet despite the low sample size, significant differences between groups were evidenced with satisfactory statistical power between 97% and 100% for most multiple comparisons. This is probably due to the accuracy of qPCR results obtained with the chosen normalization strategy. Secondly, Western blot analyses failed to detect free forms of TIMPs, active MMP2 or full-length MMP14. This might be due to the lack of sensitivity of the technique as well as possible post-translational modifications of the target proteins occurring during the rabbit lung development. As the present study was not designed to assess the MMP/TIMP balance, there was regrettably not enough material for additional and more sensitive experiments regarding MMP and TIMP activities, such as zymography, reverse zymography or activity assays. Thirdly, the use of predicted rabbit or human nucleic acid sequences could not be avoided for primer design. In spite of greater phylogenetical similarities with humans than rodents, rabbit models have indeed some limitations due the poor knowledge of the rabbit genome and the paucity of commercially available immunologic reagents.

Despite the aforementioned limitations, our results based on the rabbit model for CDH suggest that the mechanisms by which experimental TO improved alveolar formation were at least in part linked to mechanotransduction-mediated expressions of genes related to the matrix environment. New molecular targets for TO were identified in hypoplastic lungs and their expressions were related to fetal lung growth. Yet the exact meaning of such translational changes remains mainly speculative and confirmational studies are advocated. The present study also provides further evidence that mechanical stretch could influence HKG stability, and proposes a normalization strategy for pulmonary gene expression analysis in the fetal rabbit model for CDH after TO.

## Supporting Information

Figure S1 Melting curves of amplified products corresponding to the newly designed primer pairs. Graphs show a single peak of a specific product at high temperature (> 80°C) with nothing or very little detected in no-template controls A. ITGA6. **B**. ITGB1. **C**. MMP2. **D**. MMP14. **E**. TIMP1. **F**. TIMP2.
(TIF)

Figure S2 Agarose gel electrophoresis of amplified products corresponding to the newly designed primer pairs. Single bands were shown at the expected size.
(TIF)

Figure S3 Transcription profiles of the ten candidate housekeeping genes. Graphs represent the mean raw qPCR cycle threshold (Cq) values obtained in the fetal rabbit model. Error bars indicate the standard deviation. **A**. Mean Cq values in all lungs combined (*n* = 43), unventilated lungs (*n* = 21), and ventilated lungs (*n* = 22). **B**. Mean Cq values in the different experimental groups, with 8 to 9 animals per group. Non-operated, untouched fetuses; SHAM, sham-operated fetuses; DH, diaphragmatic hernia fetuses; DH+ TO, DH fetuses with tracheal occlusion; TO, sham DH fetuses with TO.
(TIF)

Figure S4 Selection of stable housekeeping genes for normalization of qPCR studies in the fetal rabbit model. Graphs represent average expression stability values (M) during stepwise exclusion calculated by *genorm*. The cut-off value for a stable gene is less than 0.5, with the lowest value for the most stable housekeeping gene. M values were below 0.5 for all tested genes. Identification of the ten candidate housekeeping genes according to M values is detailed in Table S6. **A**. M values in all lungs combined (*n* = 43), unventilated lungs (*n* = 21), and ventilated lungs (*n* = 22). **B**. M values for different compilations of experimental groups, with 8 to 9 animals per study group. SHAM, sham-operated fetuses; DH, diaphragmatic hernia fetuses; DH+ TO, DH fetuses with tracheal occlusion; TO, sham DH fetuses with TO.
(TIF)

Figure S5 Optimal number of stable housekeeping genes for normalization of qPCR studies in the fetal rabbit model. Graphs represent pair-wise variations (V) calculated by *genorm* by comparisons of the normalization factors obtained from an increasing number of genes. The cut-off value for an acceptable combination of genes is less than 0.15. The optimal number of genes was 2, as normalization factors based on the 2 most stable genes were far below 0.15 and would not change with the addition of a third gene. **A**. V values for all samples combined (*n* = 43), unventilated samples (*n* = 21), and ventilated samples (*n* = 22). **B**. V values for different compilations of experimental groups, with 8 to 9 animals per study group. SHAM, sham-operated fetuses; DH, diaphragmatic hernia fetuses; DH+ TO, DH fetuses with tracheal occlusion; TO, sham DH fetuses with TO.
(TIF)

Figure S6 Normalized expression of alveolarization genes with different normalization strategies in the fetal rabbit model for CDH undergoing TO. Mean Cq values were converted into linear values according to the efficiency corrected model, and normalized according to the best combination of housekeeping genes as suggested by *genorm* (SDHA + TOP1) or different single housekeeping genes (ACTB, HMBS, ATP5B, SDHA or TOP1). Error bars illustrate the standard error of the mean, with 4 to 5 animals per study group. One-way ANOVA with Bonferroni correction was used for comparisons between surgical groups (SHAM, DH, DH+ TO, and TO) and unpaired Student’s t-test for comparisons between non-operated and SHAM fetuses. SHAM, sham-operated fetuses; DH, diaphragmatic hernia fetuses; DH+ TO, DH fetuses with tracheal occlusion; TO, sham DH fetuses with TO. ^a^ *P* < 0.05 vs SHAM; ^b^ *P* < 0.05 vs DH; ^c^ *P* < 0.05 vs non-operated; ^d^overall *P* = 0.053. A.U. = arbitrary unit.
(TIF)

Figure S7 Western blot detection for MMP14 in lung tissue homogenates from non-operated, SHAM, DH, DH+ TO, and TO fetuses. Only a shedding fragment at approximately 22 kDa was visualized in each group. GAPDH was used as loading control. SHAM, sham-operated fetuses; DH, diaphragmatic hernia fetuses; DH+ TO, DH fetuses with tracheal occlusion; TO, sham DH fetuses with TO.
(TIF)

Figure S8 Localization of MMPs and TIMPs in lung airways and vasculature in the fetal rabbit model for CDH undergoing TO. Representative images of immunohistochemistry for MMP2 (**A**–**E**), MMP14 (**F**–**J**), TIMP1 (**K**–**O**), and TIMP2 (**P**–**T**) in non-operated (**A**, **F**, **Q**, **P**), SHAM (**B**, **G**, **L**, **K**), DH (**C**, **H**, **M**, **R**), DH+ TO (**D**, **I**, **N**, **S**), and TO lungs (**E**, **J**, **O**, **T**). No apparent differences in staining pattern for MMP2, MMP14, TIMP1 and TIMP2 were observed between the study groups. The epithelium of bronchi and bronchioles displayed a diffuse cytoplasmic immunoreactivity for MMP2, MMP14, and TIMP2 (high-magnification inserts). Moreover the apical membrane of the bronchial and bronchiolar epithelium was stained for TIMP1 (high-magnification inserts), but an artifactual staining was not excluded for certain. Regarding the lung vasculature MMP2 but not MMP14 was located in the pulmonary arteries with a strong immune reaction in the adventitia (solid arrowheads) and a faint staining of the media (open arrowheads). TIMP2 and TIMP1 were respectively localized in the adventitia (solid arrowheads) and the endothelium (arrows) of pulmonary arteries. Scale bar = 50 µm in A through T.
(TIF)

Table S1 Name and function of candidate housekeeping genes. (DOC)

Table S2 Sequences of primers previously designed for candidate housekeeping genes. (DOC)

Table S3 Sequences of primers previously designed for alveolarization genes. (DOC)

Table S4 MIQE checklist. (XLS)

Table S5 Bivariate Pearson’s correlation coefficients between the ten housekeeping genes based on raw Cq values obtained for all samples (*n* = 43). (DOC)

Table S6 Ranking of the ten candidate housekeeping genes according to their expression stability in different compilations of groups. Average expression stability (M) calculated by genorm decreases from top to bottom. All tested genes were stably expressed with M value less than 0.5. In all set-ups, HMBS was the least stable gene (highest M). RPLP0, SDHA, and TOP1 were the most stable genes in almost all experimental set-ups (lowest M). Data provided by M analysis were confirmed by the CV of the normalized HKG relative quantities calculated in all experimental set-ups, indicating acceptable values below 0.2 for RPLP0 (between 0.097 and 0.187), ATP5B (between 0.121 and 0.169), TOP1 (between 0.128 and 0.187), and SDHA (between 0.071 and 0.101), and suboptimal values for HMBS (between 0.362 and 0.495), and to a lesser extent for ACTB (between 0.173 and 0.339), HPRT (between 0.187 and 0.298), PGK1 (between 0.160 and 0.238), and GAPDH (between 0.195 and 0.227). SHAM, sham-operated fetuses; DH, diaphragmatic hernia fetuses; DH+ TO, DH fetuses with tracheal occlusion; TO, sham DH fetuses with TO. M min, minimal M value; M max, maximal M value.
(DOC)

Text S1 BLAST analysis against the whole database (mammal species) for the newly designed primer pairs. (DOC)
