# Supplementary figures and images for: Alveolarization Genes Modulated by Fetal Tracheal Occlusion in the Rabbit Model for Congenital Diaphragmatic Hernia: A Randomized Study
Source: PLoS One. 2013 Jul 1;8(7):e69210. doi: 10.1371/journal.pone.0069210 (PMC3698086; doi:10.1371/journal.pone.0069210)

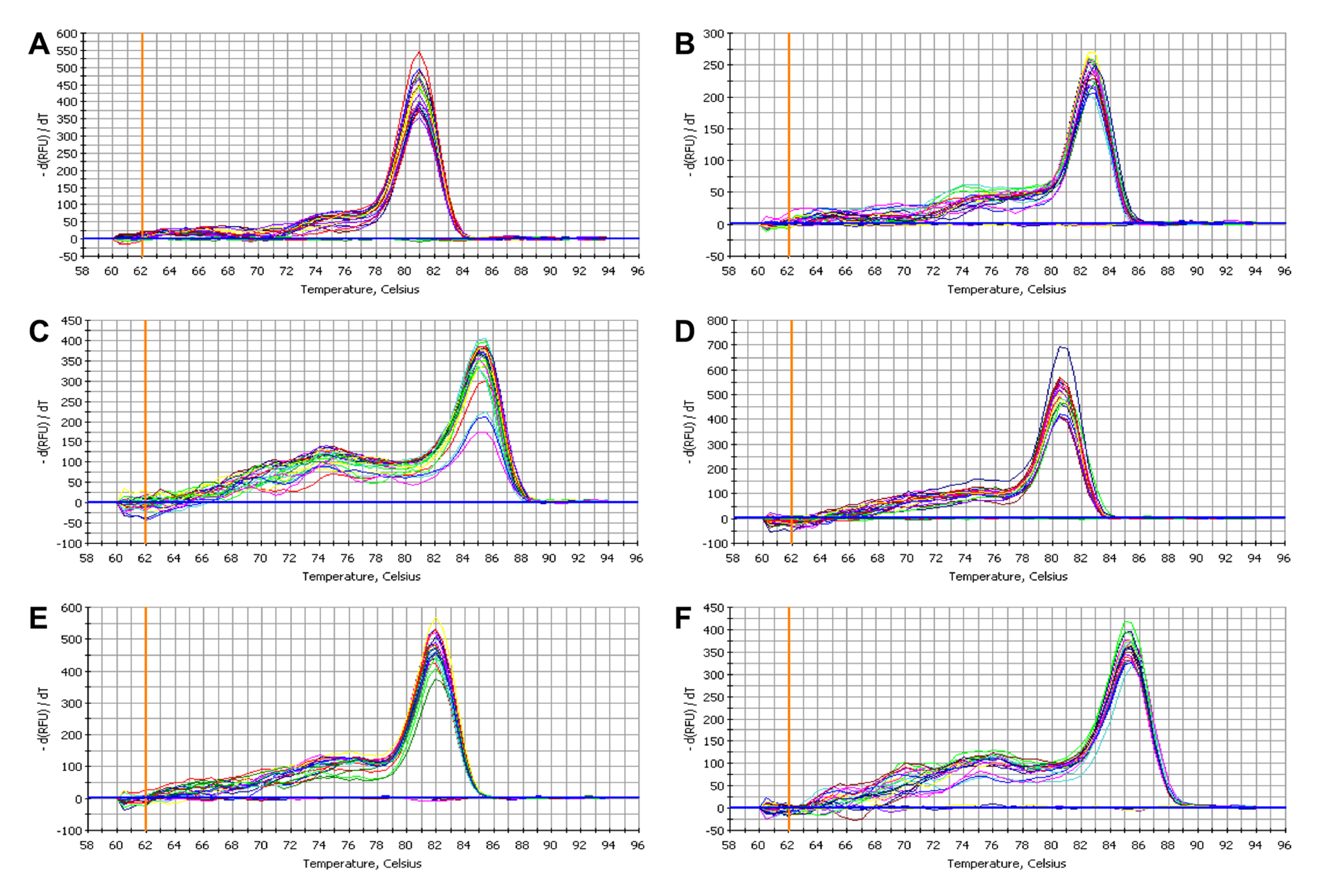

Supplement: Figure S1 — Graphs show a single peak of a specific product at high temperature (> 80°C) with nothing or very little detected in no-template controls A. ITGA6. B. ITGB1. C. MMP2. D. MMP14. E. TIMP1. F. TIMP2. (TIF) [file pone.0069210.s001.tif]

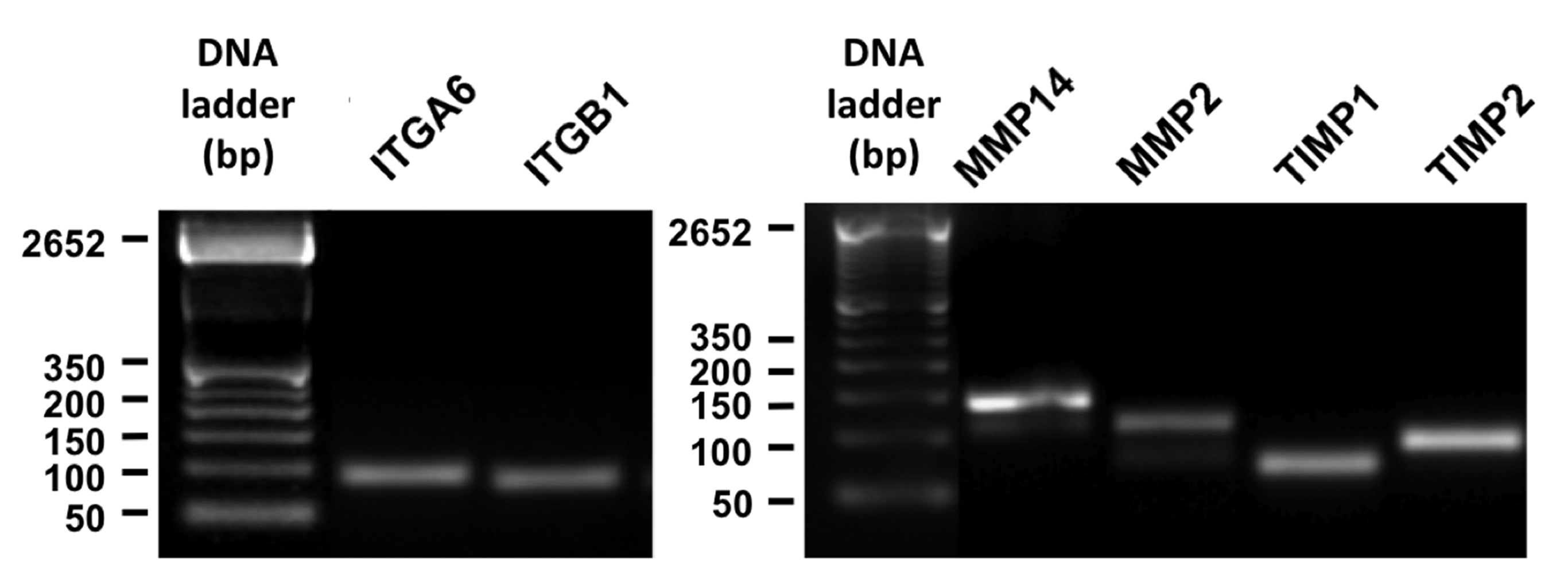

Supplement: Figure S2 — Single bands were shown at the expected size. (TIF) [file pone.0069210.s002.tif]

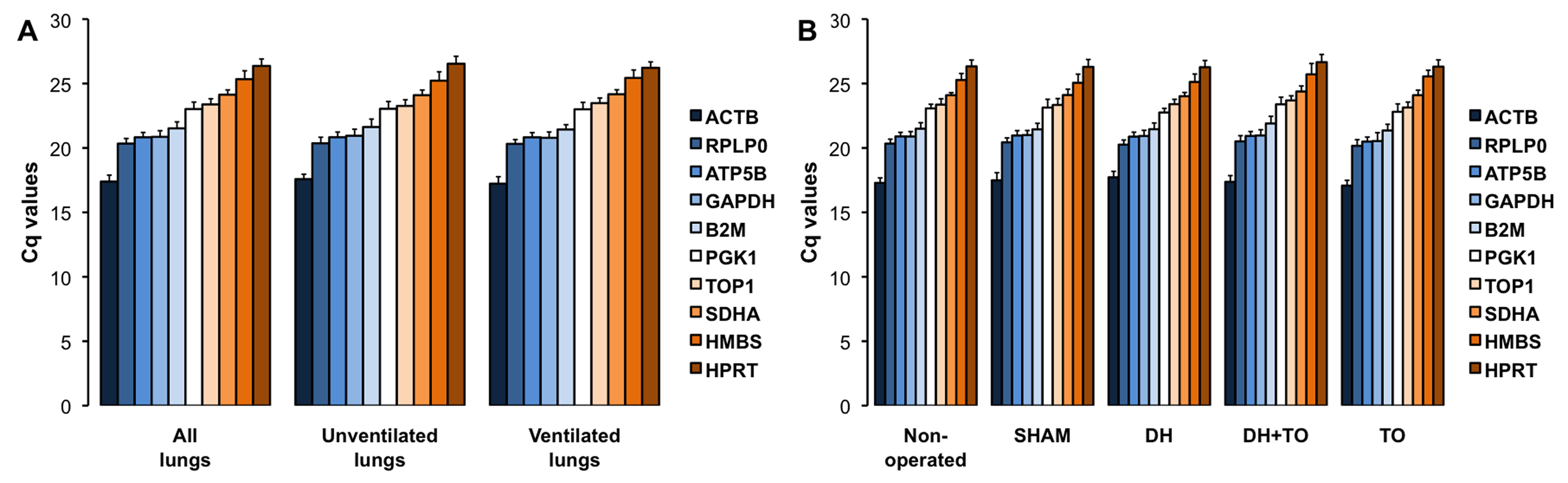

Supplement: Figure S3 — Graphs represent the mean raw qPCR cycle threshold (Cq) values obtained in the fetal rabbit model. Error bars indicate the standard deviation. A. Mean Cq values in all lungs combined (n = 43), unventilated lungs (n = 21), and ventilated lungs (n = 22). B. Mean Cq values in the different experimental groups, with 8 to 9 animals per group. Non-operated, untouched fetuses; SHAM, sham-operated fetuses; DH, diaphragmatic hernia fetuses; DH+ TO, DH fetuses with tracheal occlusion; TO, sham DH fetuses with TO. (TIF) [file pone.0069210.s003.tif]

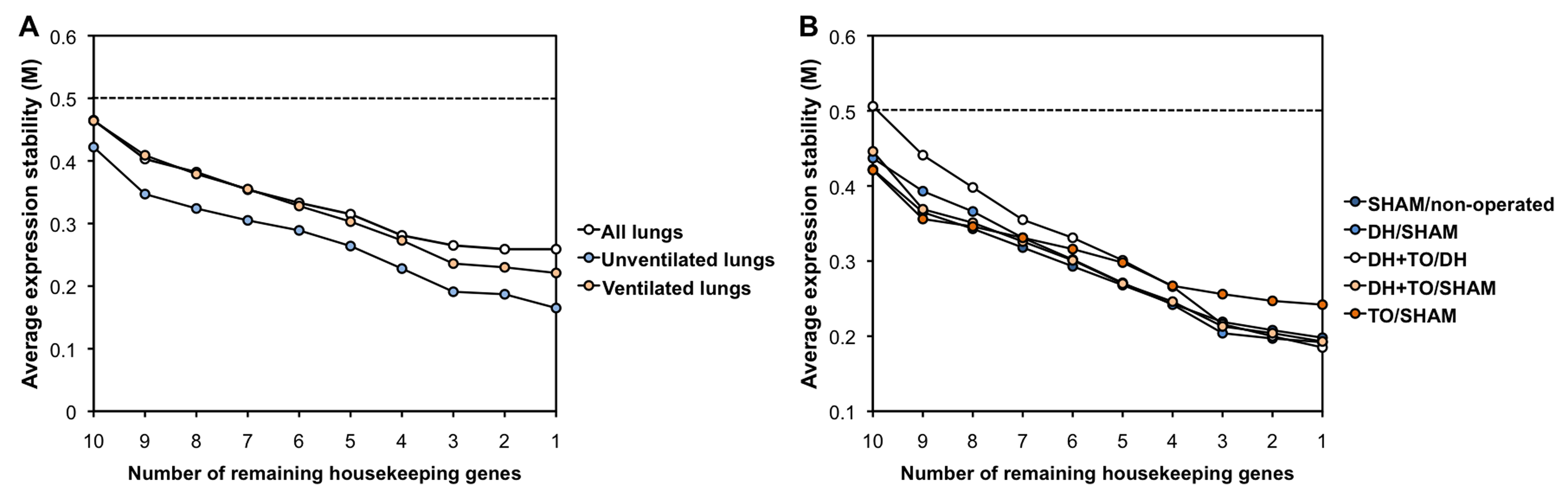

Supplement: Figure S4 — Graphs represent average expression stability values (M) during stepwise exclusion calculated by genorm. The cut-off value for a stable gene is less than 0.5, with the lowest value for the most stable housekeeping gene. M values were below 0.5 for all tested genes. Identification of the ten candidate housekeeping genes according to M values is detailed in Table S6. A. M values in all lungs combined (n = 43), unventilated lungs (n = 21), and ventilated lungs (n = 22). B. M values for different compilations of experimental groups, with 8 to 9 animals per study group. SHAM, sham-operated fetuses; DH, diaphragmatic hernia fetuses; DH+ TO, DH fetuses with tracheal occlusion; TO, sham DH fetuses with TO. (TIF) [file pone.0069210.s004.tif]

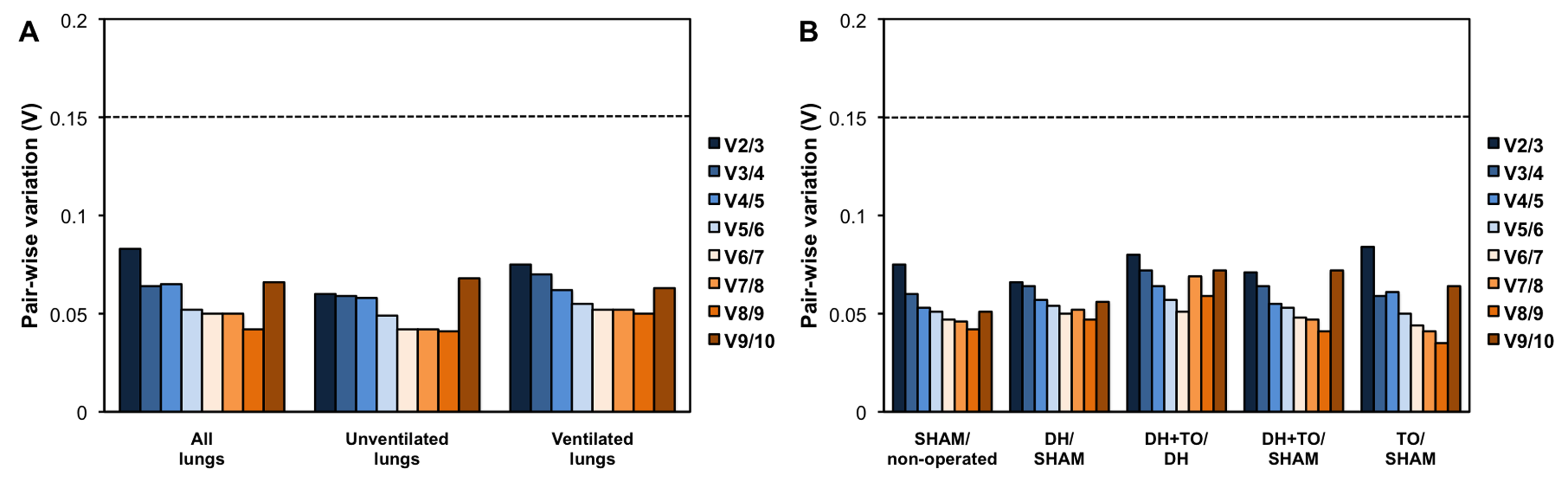

Supplement: Figure S5 — Graphs represent pair-wise variations (V) calculated by genorm by comparisons of the normalization factors obtained from an increasing number of genes. The cut-off value for an acceptable combination of genes is less than 0.15. The optimal number of genes was 2, as normalization factors based on the 2 most stable genes were far below 0.15 and would not change with the addition of a third gene. A. V values for all samples combined (n = 43), unventilated samples (n = 21), and ventilated samples (n = 22). B. V values for different compilations of experimental groups, with 8 to 9 animals per study group. SHAM, sham-operated fetuses; DH, diaphragmatic hernia fetuses; DH+ TO, DH fetuses with tracheal occlusion; TO, sham DH fetuses with TO. (TIF) [file pone.0069210.s005.tif]

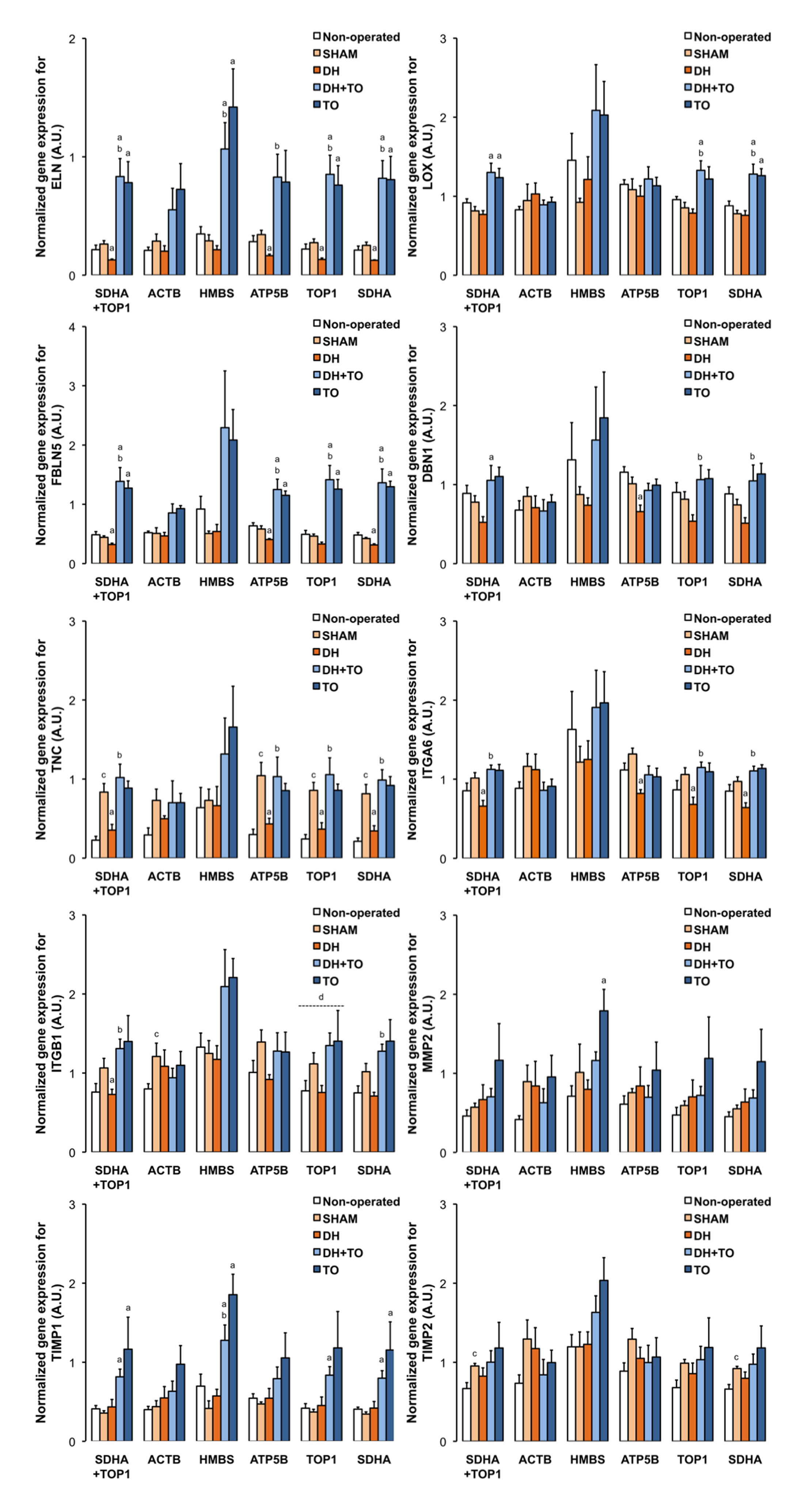

Supplement: Figure S6 — Mean Cq values were converted into linear values according to the efficiency corrected model, and normalized according to the best combination of housekeeping genes as suggested by genorm (SDHA + TOP1) or different single housekeeping genes (ACTB, HMBS, ATP5B, SDHA or TOP1). Error bars illustrate the standard error of the mean, with 4 to 5 animals per study group. One-way ANOVA with Bonferroni correction was used for comparisons between surgical groups (SHAM, DH, DH+ TO, and TO) and unpaired Student’s t-test for comparisons between non-operated and SHAM fetuses. SHAM, sham-operated fetuses; DH, diaphragmatic hernia fetuses; DH+ TO, DH fetuses with tracheal occlusion; TO, sham DH fetuses with TO. a P < 0.05 vs SHAM; b P < 0.05 vs DH; c P < 0.05 vs non-operated; doverall P = 0.053. A.U. = arbitrary unit. (TIF) [file pone.0069210.s006.tif]

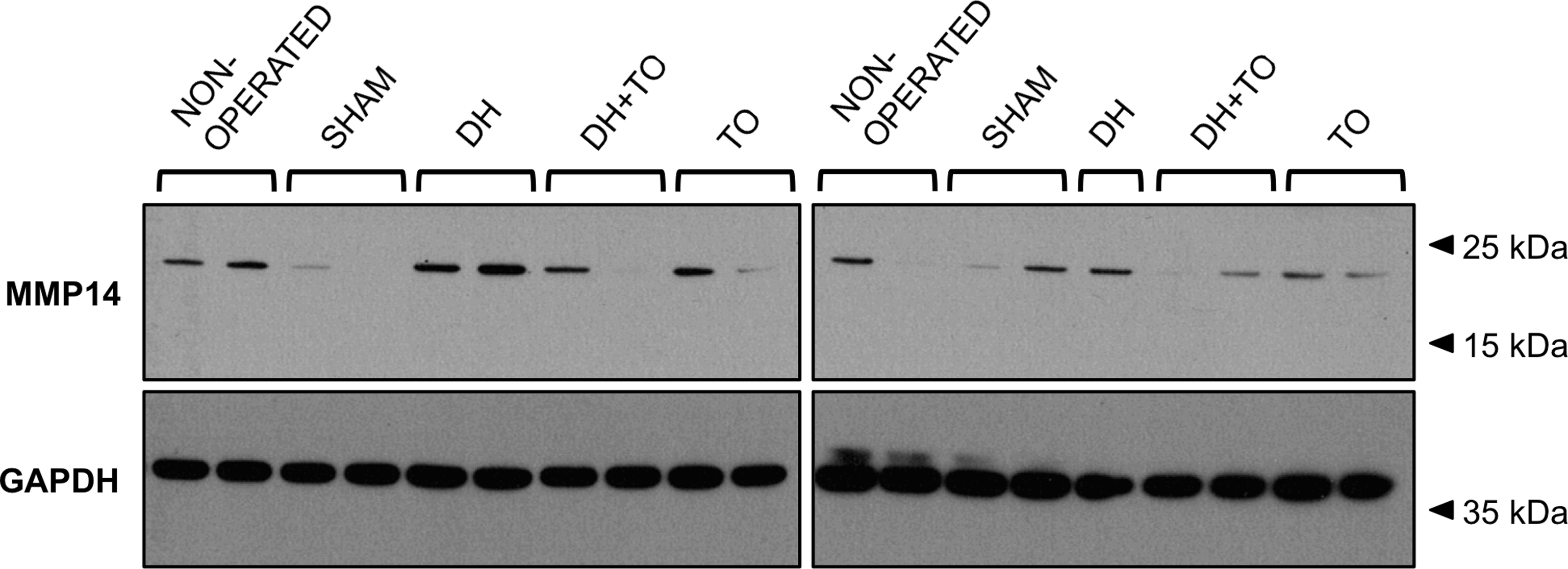

Supplement: Figure S7 — Only a shedding fragment at approximately 22 kDa was visualized in each group. GAPDH was used as loading control. SHAM, sham-operated fetuses; DH, diaphragmatic hernia fetuses; DH+ TO, DH fetuses with tracheal occlusion; TO, sham DH fetuses with TO. (TIF) [file pone.0069210.s007.tif]

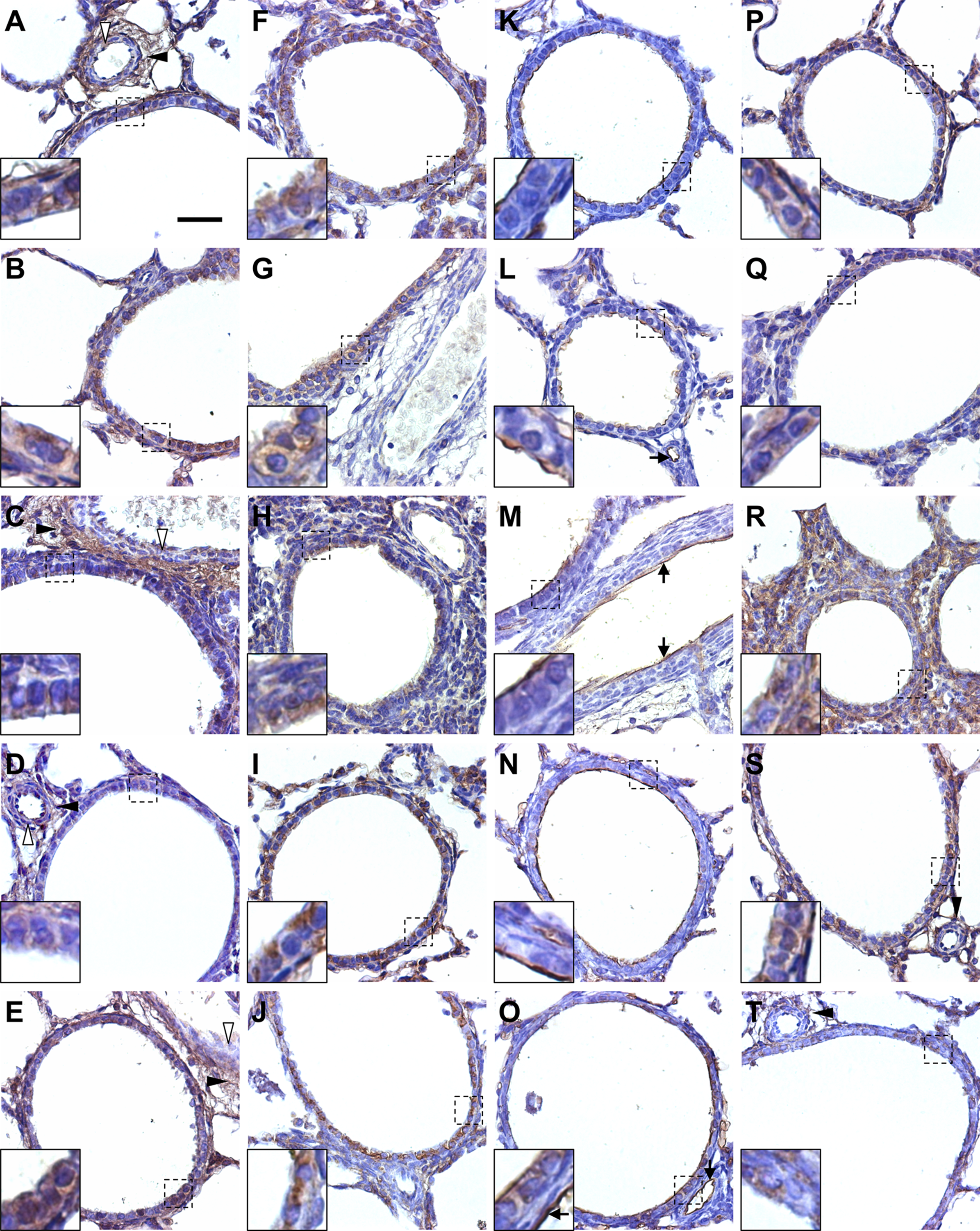

Supplement: Figure S8 — Representative images of immunohistochemistry for MMP2 (A–E), MMP14 (F–J), TIMP1 (K–O), and TIMP2 (P–T) in non-operated (A, F, Q, P), SHAM (B, G, L, K), DH (C, H, M, R), DH+ TO (D, I, N, S), and TO lungs (E, J, O, T). No apparent differences in staining pattern for MMP2, MMP14, TIMP1 and TIMP2 were observed between the study groups. The epithelium of bronchi and bronchioles displayed a diffuse cytoplasmic immunoreactivity for MMP2, MMP14, and TIMP2 (high-magnification inserts). Moreover the apical membrane of the bronchial and bronchiolar epithelium was stained for TIMP1 (high-magnification inserts), but an artifactual staining was not excluded for certain. Regarding the lung vasculature MMP2 but not MMP14 was located in the pulmonary arteries with a strong immune reaction in the adventitia (solid arrowheads) and a faint staining of the media (open arrowheads). TIMP2 and TIMP1 were respectively localized in the adventitia (solid arrowheads) and the endothelium (arrows) of pulmonary arteries. Scale bar = 50 µm in A through T. (TIF) [file pone.0069210.s008.tif]
