# Supplementary material for: Alveolarization Genes Modulated by Fetal Tracheal Occlusion in the Rabbit Model for Congenital Diaphragmatic Hernia: A Randomized Study
Source: PLoS One. 2013 Jul 1;8(7):e69210. doi: 10.1371/journal.pone.0069210 (PMC3698086; doi:10.1371/journal.pone.0069210)
Supplement: Table S1 — (DOC) [file pone.0069210.s009.doc]

**Table S1. Name and function of candidate housekeeping genes.**

| **Symbol** | **Name** | **Function** |
| --- | --- | --- |
| ACTB | ß-actin | Cytoskeleletal structural protein |
| ATP5B | Mitochondrial ATP synthase, subunit ß | Catalysis of ATP synthesis |
| B2M | ß2-microglobulin | Presentation of antigens to the immune system |
| GAPDH | Glyceraldehyde 3-phosphate dehydrogenase | Glycolysis and gluconeogenesis |
| HMBS | Hydroxymethylbilane synthase | Catalysis of heme biosynthesis |
| HPRT | Hypoxanthine guanine phosphoribosyl transferase | Purine synthesis in salvage pathway |
| PGK1 | Phosphoglycerate kinase 1 | Glycolysis |
| RPLP0 | Ribosomal protein, large, P0 | Ribosomal structural protein |
| SDHA | Succinate dehydrogenase complex, subunit A | Citric Acid Cycle and Respiratory chain |
| TOP1 | DNA topoisomerase 1 | Control of transcription |
