# Supplementary material for: Alveolarization Genes Modulated by Fetal Tracheal Occlusion in the Rabbit Model for Congenital Diaphragmatic Hernia: A Randomized Study
Source: PLoS One. 2013 Jul 1;8(7):e69210. doi: 10.1371/journal.pone.0069210 (PMC3698086; doi:10.1371/journal.pone.0069210)
Supplement: Table S2 — (DOC) [file pone.0069210.s010.doc]

**Table S**2. Sequences of primers previously designed for candidate housekeeping genes.

| **Gene (accession number)** | **Primer sequences – 5’ to 3’** | **T_m_ (°C)** | **E** | **R^2^** |
| --- | --- | --- | --- | --- |
| ACTB (NM_001101683) | F: GATCTGGCACCACACCTTCT | 60.12 | 2.037 | 0.994 |
|  | R: TGATCTGGGTCATCTTCTCG | 58.75 |  |  |
|  | Amplicon size: 116 bp |  |  |  |
| ATP5B (XM_002711084) | F: GAGGTCCCATCAAAACCAAA | 59.77 | 1.967 | 0.998 |
|  | R: TTTCCTGCTCCACACTCATCT | 59.86 |  |  |
|  | Amplicon size: 81 bp |  |  |  |
| B2M (XM_002717921) | F: CGCCCCAGATTGATATTGAG | 60.43 | 2.017 | 0.999 |
|  | R: GGACCAGGAGATAGAAAGACCA | 59.58 |  |  |
|  | Amplicon size: 105 bp |  |  |  |
| GAPDH (NM_001082253) | F: AGGTCGGAGTGAACGGATT | 59.52 | 1.990 | 1.000 |
|  | R: ATGGCGACAACATCCACTTT | 60.38 |  |  |
|  | Amplicon size: 85 bp |  |  |  |
| HMBS (XM_002722723) | F: GGCAACGCTGAAAACCTTAT | 59.23 | 2.010 | 0.998 |
|  | R: AGGCTCTTCTCCCCAATCTT | 59.28 |  |  |
|  | Amplicon size: 111 bp |  |  |  |
| HPRT (NM_001105671) | F: GGCAAAACAATGCAGACCTT | 60.12 | 1.990 | 0.999 |
|  | R: CTTCGAGGGGTCCTTTTCAC | 60.99 |  |  |
|  | Amplicon size: 95 bp |  |  |  |
| PGK1 (XM_002709000) | F: CTAGGCGGAGCTAAAGTTGC | 59.27 | 2.012 | 0.997 |
|  | R: AGCCATTCCACCACCAATAA | 60.19 |  |  |
|  | Amplicon size: 90 bp |  |  |  |
| RPLP0 (XM_002719794) | F: ACCTCCTTTTTCCAGGCTTT | 59.21 | 2.023 | 0.996 |
|  | R: GCTCCCACTTTGTCTCCAGT | 59.30 |  |  |
|  | Amplicon size: 104 bp |  |  |  |
| SDHA (XM_002723194) | F: ATCTATCAGCGTGCGTTCG | 59.99 | 1.988 | 0.996 |
|  | R: ATCAGCCACACAGCAGCAT | 60.45 |  |  |
|  | Amplicon size: 81 bp |  |  |  |
| TOP1 (NM_001101683) | F: GCAGGCAATGAGAAGGAAGA | 60.48 | 1.989 | 0.998 |
|  | R: CACGTACTCCTGACCATCCA | 59.55 |  |  |
|  | Amplicon size: 108 bp |  |  |  |

F, forward primer; R, reverse primer; T_m_, melting temperature; bp, number of base pairs; E, real-time PCR efficiency; R^2^, coefficient of determination.
