# Supplementary material for: Alveolarization Genes Modulated by Fetal Tracheal Occlusion in the Rabbit Model for Congenital Diaphragmatic Hernia: A Randomized Study
Source: PLoS One. 2013 Jul 1;8(7):e69210. doi: 10.1371/journal.pone.0069210 (PMC3698086; doi:10.1371/journal.pone.0069210)
Supplement: Table S3 — (DOC) [file pone.0069210.s011.doc]

**Table S3. Sequences of primers previously designed for alveolarization genes.**

| **Gene (accession number)** | **Primer sequences – 5’ to 3’** | **T_m_ (°C)** | **E** | **R^2^** |
| --- | --- | --- | --- | --- |
| ELN (XM_002721971) | F: AGCCAAATACGGTGCTGCT | 60.81 | 1.931 | 0.979 |
|  | R: CACCTGGGTAAATGGGAGAC | 59.26 |  |  |
|  | Amplicon size: 110 bp |  |  |  |
| LOX (XM_002710146) | F: CCCCCAACGAGTGAAAAAC | 60.34 | 2.026 | 0.995 |
|  | R: ATGCTGTGGTAATGCTGGTG | 59.60 |  |  |
|  | Amplicon size: 99 bp |  |  |  |
| FBLN5 (XM_002719596) | F: CAACTACCCCACGATTTCCA | 60.74 | 2.025 | 0.998 |
|  | R: TCCACATCCACACACTGGTT | 59.85 |  |  |
|  | Amplicon size: 81 bp |  |  |  |
| TNC (XM_002720513) | F: TGAACTCTCCTACGGCATCA | 59.39 | 2.020 | 0.998 |
|  | R: TCATACTCGGTGTCAGGCTTC | 60.27 |  |  |
|  | Amplicon size: 111 bp |  |  |  |
| DBN1 (NM_001082149) | F: GGAAATGAAACGCATCAACC | 60.32 | 1.974 | 0.997 |
|  | R: CTTCTTCCGCTCCTCTTCCT | 60.09 |  |  |
|  | Amplicon size: 85 bp |  |  |  |

F, forward primer; R, reverse primer; T_m_, melting temperature; bp, number of base pairs; E, real-time PCR efficiency; R^2^, coefficient of determination.
