# Supplementary material for: Alveolarization Genes Modulated by Fetal Tracheal Occlusion in the Rabbit Model for Congenital Diaphragmatic Hernia: A Randomized Study
Source: PLoS One. 2013 Jul 1;8(7):e69210. doi: 10.1371/journal.pone.0069210 (PMC3698086; doi:10.1371/journal.pone.0069210)
Supplement: Table S5 — (DOC) [file pone.0069210.s013.doc]

**Table S5. Bivariate Pearson’s correlation coefficients between the ten housekeeping genes based on raw Cq values obtained for all samples (*n*** = 43).

| Gene | ACTB | ATP5B | B2M | GAPDH | HMBS | HPRT | PGK1 | RPLP0 | SDHA | TOP1 |
| --- | --- | --- | --- | --- | --- | --- | --- | --- | --- | --- |
| ACTB | / | / | / | / | / | / | / | / | / | / |
| ATP5B | 0.724^a^ | / | / | / | / | / | / | / | / | / |
| B2M | 0.533^a^ | 0.564^a^ | / | / | / | / | / | / | / | / |
| GAPDH | 0.523^a^ | 0.701^a^ | 0.508^a^ | / | / | / | / | / | / | / |
| HMBS | 0.033 | 0.074 | 0.311^c^ | 0.123 | / | / | / | / | / | / |
| HPRT | 0.446^b^ | 0.445^b^ | 0.751^a^ | 0.557^a^ | 0.059 | / | / | / | / | / |
| PGK1 | 0.447^b^ | 0.739^a^ | 0.565^a^ | 0.788^a^ | 0.254 | 0.582^a^ | / | / | / | / |
| RPLP0 | 0.523^a^ | 0.661^a^ | 0.697^a^ | 0.758^a^ | 0.346^c^ | 0.516^a^ | 0.767^a^ | / | / | / |
| SDHA | 0.511^a^ | 0.715^a^ | 0.736^a^ | 0.620^a^ | 0.512^a^ | 0.610^a^ | 0.670^a^ | 0.810^a^ | / | / |
| TOP1 | 0.514^a^ | 0.782^a^ | 0.655^a^ | 0.543^a^ | 0.398^b^ | 0.438^b^ | 0.670^a^ | 0.693^a^ | 0.834^a^ | / |

^a^*P* < 0.001; ^b^*P* < 0.01; ^c^*P* < 0.05.
