# Supplementary material for: Alveolarization Genes Modulated by Fetal Tracheal Occlusion in the Rabbit Model for Congenital Diaphragmatic Hernia: A Randomized Study
Source: PLoS One. 2013 Jul 1;8(7):e69210. doi: 10.1371/journal.pone.0069210 (PMC3698086; doi:10.1371/journal.pone.0069210)
Supplement: Table S6 — Average expression stability (M) calculated by genorm decreases from top to bottom. All tested genes were stably expressed with M value less than 0.5. In all set-ups, HMBS was the least stable gene (highest M). RPLP0, SDHA, and TOP1 were the most stable genes in almost all experimental set-ups (lowest M). Data provided by M analysis were confirmed by the CV of the normalized HKG relative quantities calculated in all experimental set-ups, indicating acceptable values below 0.2 for RPLP0 (between 0.097 and 0.187), ATP5B (between 0.121 and 0.169), TOP1 (between 0.128 and 0.187), and SDHA (between 0.071 and 0.101), and suboptimal values for HMBS (between 0.362 and 0.495), and to a lesser extent for ACTB (between 0.173 and 0.339), HPRT (between 0.187 and 0.298), PGK1 (between 0.160 and 0.238), and GAPDH (between 0.195 and 0.227). SHAM, sham-operated fetuses; DH, diaphragmatic hernia fetuses; DH+ TO, DH fetuses with tracheal occlusion; TO, sham DH fetuses with TO. M min, minimal M value; M max, maximal M value. (DOC) [file pone.0069210.s014.doc]

**Table S6**. Ranking of the ten candidate housekeeping genes according to their expression stability in different compilations of groups.

| **Set-ups** | **All fetuses** | **Unventilated fetuses** | **Ventilated fetuses** | **SHAM and unoperated fetuses** | **DH and SHAM fetuses** | **DH+TO and DH fetuses** | **DH+TO and SHAM fetuses** | **TO and SHAM fetuses** |
| --- | --- | --- | --- | --- | --- | --- | --- | --- |
|  | *n* = 43 | *n* = 21 | *n* = 22 | *n* = 17 | *n* = 18 | *n* = 17 | *n* = 17 | *n* = 18 |
| **M max** | **0.464** | **0.416** | **0.464** | **0.429** | **0.436** | **0.507** | **0.446** | **0.449** |
|  | HMBS | HMBS | HMBS | HMBS | HMBS | HMBS | HMBS | HMBS |
|  | ACTB | HPRT | ACTB | B2M | ACTB | ACTB | HPRT | PGK1 |
|  | HPRT | GAPDH | HPRT | HPRT | HPRT | HPRT | B2M | GAPDH |
|  | B2M | PGK1 | PGK1 | ACTB | PGK1 | PGK1 | ACTB | ACTB |
|  | PGK1 | RPLP0 | B2M | TOP1 | B2M | GAPDH | PGK1 | HPRT |
|  | GAPDH | B2M | GAPDH | PGK1 | GAPDH | ATP5B | GAPDH | B2M |
|  | RPLP0 | SDHA | ATP5B | GAPDH | RPLP0 | B2M | ATP5B | RPLP0 |
|  | ATP5B | ATP5B | RPLP0 | RPLP0 | TOP1 | RPLP0 | RPLP0 | ATP5B |
|  | SDHA | ACTB | TOP1 | ATP5B | ATP5B | TOP1 | TOP1 | SDHA |
|  | TOP1 | TOP1 | SDHA | SDHA | SDHA | SDHA | SDHA | TOP1 |
| **M min** | **0.261** | **0.173** | **0.221** | **0.198** | **0.192** | **0.184** | **0.193** | **0.196** |
