# Supplementary material for: Alveolarization Genes Modulated by Fetal Tracheal Occlusion in the Rabbit Model for Congenital Diaphragmatic Hernia: A Randomized Study
Source: PLoS One. 2013 Jul 1;8(7):e69210. doi: 10.1371/journal.pone.0069210 (PMC3698086; doi:10.1371/journal.pone.0069210)
Supplement: Text S1 — (DOC) [file pone.0069210.s015.doc]

**Text S1. BLAST analysis against the whole database (mammal species) for the newly designed primer pairs.**

**Target templates; primers for Integrin-α6**

[XM_002712183.1](http://www.ncbi.nlm.nih.gov/entrez/viewer.fcgi?db=nucleotide&id=291391740) PREDICTED: Oryctolagus cuniculus integrin, alpha 6 (ITGA6), mRNA

product length = 96

Forward primer 1 GTGACTGTGTTTCCCTCCAAG 21

Template 2983 ..................... 3003

Reverse primer 1 GCAAGCATCAAAATCCCAAC 20

Template 3077 .................... 3058

[NM_001258117.1](http://www.ncbi.nlm.nih.gov/entrez/viewer.fcgi?db=nucleotide&id=384475800) Macaca mulatta integrin, alpha 6 (ITGA6), mRNA

product length = 95

Forward primer 1 GTGACTGTGTTTCCCTCCAAG 21

Template 3188 .................A... 3208

Reverse primer 1 GCAAGCATCAAAATCCCAAC 20

Template 3282 ..................G. 3263

[NM_001109981.1](http://www.ncbi.nlm.nih.gov/entrez/viewer.fcgi?db=nucleotide&id=158341671) Bos taurus integrin, alpha 6 (ITGA6), mRNA

product length = 95

Forward primer 1 GTGACTGTGTTTCCCTCCAAG 21

Template 2995 .................A... 3015

Reverse primer 1 GCAAGCATCAAAATCCCAAC 20

Template 3089 C.................G. 3070

[NM_000210.2](http://www.ncbi.nlm.nih.gov/entrez/viewer.fcgi?db=nucleotide&id=119395741) Homo sapiens integrin, alpha 6 (ITGA6), transcript variant 2, mRNA

product length = 95

Forward primer 1 GTGACTGTGTTTCCCTCCAAG 21

Template 3198 .................A... 3218

Reverse primer 1 GCAAGCATCAAAATCCCAAC 20

Template 3292 ...........G......G. 3273

[NM_001079818.1](http://www.ncbi.nlm.nih.gov/entrez/viewer.fcgi?db=nucleotide&id=119395739) Homo sapiens integrin, alpha 6 (ITGA6), transcript variant 1, mRNA

product length = 95

Forward primer 1 GTGACTGTGTTTCCCTCCAAG 21

Template 3198 .................A... 3218

Reverse primer 1 GCAAGCATCAAAATCCCAAC 20

Template 3292 ...........G......G. 3273

[XM_003921869.1](http://www.ncbi.nlm.nih.gov/entrez/viewer.fcgi?db=nucleotide&id=403258767) PREDICTED: Saimiri boliviensis boliviensis integrin, alpha 6, transcript variant 3 (ITGA6), mRNA

product length = 95

Forward primer 1 GTGACTGTGTTTCCCTCCAAG 21

Template 3337 ..................... 3357

Reverse primer 1 GCAAGCATCAAAATCCCAAC 20

Template 3431 ..................G. 3412

[XM_003921868.1](http://www.ncbi.nlm.nih.gov/entrez/viewer.fcgi?db=nucleotide&id=403258765) PREDICTED: Saimiri boliviensis boliviensis integrin, alpha 6, transcript variant 2 (ITGA6), mRNA

product length = 95

Forward primer 1 GTGACTGTGTTTCCCTCCAAG 21

Template 3220 ..................... 3240

Reverse primer 1 GCAAGCATCAAAATCCCAAC 20

Template 3314 ..................G. 3295

[XM_003921867.1](http://www.ncbi.nlm.nih.gov/entrez/viewer.fcgi?db=nucleotide&id=403258763) PREDICTED: Saimiri boliviensis boliviensis integrin, alpha 6, transcript variant 1 (ITGA6), mRNA

product length = 95

Forward primer 1 GTGACTGTGTTTCCCTCCAAG 21

Template 3220 ..................... 3240

Reverse primer 1 GCAAGCATCAAAATCCCAAC 20

Template 3314 ..................G. 3295

[XM_003733166.1](http://www.ncbi.nlm.nih.gov/entrez/viewer.fcgi?db=nucleotide&id=390464382) PREDICTED: Callithrix jacchus integrin, alpha 6, transcript variant 2 (ITGA6), mRNA

product length = 95

Forward primer 1 GTGACTGTGTTTCCCTCCAAG 21

Template 3186 ..................... 3206

Reverse primer 1 GCAAGCATCAAAATCCCAAC 20

Template 3280 ..................G. 3261

[XM_002749342.2](http://www.ncbi.nlm.nih.gov/entrez/viewer.fcgi?db=nucleotide&id=390464380) PREDICTED: Callithrix jacchus integrin, alpha 6, transcript variant 1 (ITGA6), mRNA

product length = 95

Forward primer 1 GTGACTGTGTTTCCCTCCAAG 21

Template 3194 ..................... 3214

Reverse primer 1 GCAAGCATCAAAATCCCAAC 20

Template 3288 ..................G. 3269

[XM_003990863.1](http://www.ncbi.nlm.nih.gov/entrez/viewer.fcgi?db=nucleotide&id=410968849) PREDICTED: Felis catus integrin, alpha 6 (ITGA6), mRNA

product length = 95

Forward primer 1 GTGACTGTGTTTCCCTCCAAG 21

Template 2792 .................A... 2812

Reverse primer 1 GCAAGCATCAAAATCCCAAC 20

Template 2886 ..................G. 2867

[XM_001925637.4](http://www.ncbi.nlm.nih.gov/entrez/viewer.fcgi?db=nucleotide&id=350593605) PREDICTED: Sus scrofa integrin, alpha 6 (ITGA6), mRNA

product length = 95

Forward primer 1 GTGACTGTGTTTCCCTCCAAG 21

Template 1660 .................A... 1680

Reverse primer 1 GCAAGCATCAAAATCCCAAC 20

Template 1754 ..................G. 1735

[XM_003640176.1](http://www.ncbi.nlm.nih.gov/entrez/viewer.fcgi?db=nucleotide&id=359323904) PREDICTED: Canis lupus familiaris integrin alpha-6-like (LOC100856563), mRNA

product length = 95

Forward primer 1 GTGACTGTGTTTCCCTCCAAG 21

Template 2995 .................A... 3015

Reverse primer 1 GCAAGCATCAAAATCCCAAC 20

Template 3089 ..................G. 3070

[XM_004032794.1](http://www.ncbi.nlm.nih.gov/entrez/viewer.fcgi?db=nucleotide&id=426337711) PREDICTED: Gorilla gorilla gorilla integrin, alpha 6, transcript variant 3 (ITGA6), mRNA

product length = 95

Forward primer 1 GTGACTGTGTTTCCCTCCAAG 21

Template 2909 .................A... 2929

Reverse primer 1 GCAAGCATCAAAATCCCAAC 20

Template 3003 ...........G......G. 2984

[XM_004032793.1](http://www.ncbi.nlm.nih.gov/entrez/viewer.fcgi?db=nucleotide&id=426337709) PREDICTED: Gorilla gorilla gorilla integrin, alpha 6, transcript variant 2 (ITGA6), mRNA

product length = 95

Forward primer 1 GTGACTGTGTTTCCCTCCAAG 21

Template 3229 .................A... 3249

Reverse primer 1 GCAAGCATCAAAATCCCAAC 20

Template 3323 ...........G......G. 3304

[XM_004032792.1](http://www.ncbi.nlm.nih.gov/entrez/viewer.fcgi?db=nucleotide&id=426337707) PREDICTED: Gorilla gorilla gorilla integrin, alpha 6, transcript variant 1 (ITGA6), mRNA

product length = 95

Forward primer 1 GTGACTGTGTTTCCCTCCAAG 21

Template 3223 .................A... 3243

Reverse primer 1 GCAAGCATCAAAATCCCAAC 20

Template 3317 ...........G......G. 3298

[XM_004004590.1](http://www.ncbi.nlm.nih.gov/entrez/viewer.fcgi?db=nucleotide&id=426220878) PREDICTED: Ovis aries integrin, alpha 6 (ITGA6), mRNA

product length = 95

Forward primer 1 GTGACTGTGTTTCCCTCCAAG 21

Template 2791 .................A... 2811

Reverse primer 1 GCAAGCATCAAAATCCCAAC 20

Template 2885 C.................G. 2866

[XM_003309418.2](http://www.ncbi.nlm.nih.gov/entrez/viewer.fcgi?db=nucleotide&id=410035889) PREDICTED: Pan troglodytes integrin, alpha 6, transcript variant 3 (ITGA6), mRNA

product length = 95

Forward primer 1 GTGACTGTGTTTCCCTCCAAG 21

Template 2909 .................A... 2929

Reverse primer 1 GCAAGCATCAAAATCCCAAC 20

Template 3003 ...........G......G. 2984

[XM_515909.4](http://www.ncbi.nlm.nih.gov/entrez/viewer.fcgi?db=nucleotide&id=410035888) PREDICTED: Pan troglodytes integrin, alpha 6, transcript variant 4 (ITGA6), mRNA

product length = 95

Forward primer 1 GTGACTGTGTTTCCCTCCAAG 21

Template 3223 .................A... 3243

Reverse primer 1 GCAAGCATCAAAATCCCAAC 20

Template 3317 ...........G......G. 3298

[XM_003309416.2](http://www.ncbi.nlm.nih.gov/entrez/viewer.fcgi?db=nucleotide&id=410035887) PREDICTED: Pan troglodytes integrin, alpha 6, transcript variant 1 (ITGA6), mRNA

product length = 95

Forward primer 1 GTGACTGTGTTTCCCTCCAAG 21

Template 3223 .................A... 3243

Reverse primer 1 GCAAGCATCAAAATCCCAAC 20

Template 3317 ...........G......G. 3298

[XM_003824258.1](http://www.ncbi.nlm.nih.gov/entrez/viewer.fcgi?db=nucleotide&id=397507658) PREDICTED: Pan paniscus integrin, alpha 6, transcript variant 2 (ITGA6), mRNA

product length = 95

Forward primer 1 GTGACTGTGTTTCCCTCCAAG 21

Template 2915 .................A... 2935

Reverse primer 1 GCAAGCATCAAAATCCCAAC 20

Template 3009 ...........G......G. 2990

[XM_003824257.1](http://www.ncbi.nlm.nih.gov/entrez/viewer.fcgi?db=nucleotide&id=397507656) PREDICTED: Pan paniscus integrin, alpha 6, transcript variant 1 (ITGA6), mRNA

product length = 95

Forward primer 1 GTGACTGTGTTTCCCTCCAAG 21

Template 3041 .................A... 3061

Reverse primer 1 GCAAGCATCAAAATCCCAAC 20

Template 3135 ...........G......G. 3116

[XM_002812597.2](http://www.ncbi.nlm.nih.gov/entrez/viewer.fcgi?db=nucleotide&id=395732479) PREDICTED: Pongo abelii integrin, alpha 6 (ITGA6), mRNA

product length = 95

Forward primer 1 GTGACTGTGTTTCCCTCCAAG 21

Template 3472 .................A... 3492

Reverse primer 1 GCAAGCATCAAAATCCCAAC 20

Template 3566 ...........G......G. 3547

[XM_003800876.1](http://www.ncbi.nlm.nih.gov/entrez/viewer.fcgi?db=nucleotide&id=395857046) PREDICTED: Otolemur garnettii integrin, alpha 6, transcript variant 2 (ITGA6), mRNA

product length = 95

Forward primer 1 GTGACTGTGTTTCCCTCCAAG 21

Template 3312 ........A........A... 3332

Reverse primer 1 GCAAGCATCAAAATCCCAAC 20

Template 3406 ..................G. 3387

[XM_003800875.1](http://www.ncbi.nlm.nih.gov/entrez/viewer.fcgi?db=nucleotide&id=395857044) PREDICTED: Otolemur garnettii integrin, alpha 6, transcript variant 1 (ITGA6), mRNA

product length = 95

Forward primer 1 GTGACTGTGTTTCCCTCCAAG 21

Template 3195 ........A........A... 3215

Reverse primer 1 GCAAGCATCAAAATCCCAAC 20

Template 3289 ..................G. 3270

[XM_001495066.3](http://www.ncbi.nlm.nih.gov/entrez/viewer.fcgi?db=nucleotide&id=338715794) PREDICTED: Equus caballus integrin, alpha 6 (ITGA6), mRNA

product length = 95

Forward primer 1 GTGACTGTGTTTCCCTCCAAG 21

Template 2798 ..............T..A... 2818

Reverse primer 1 GCAAGCATCAAAATCCCAAC 20

Template 2892 ..................G. 2873

[XM_002927917.1](http://www.ncbi.nlm.nih.gov/entrez/viewer.fcgi?db=nucleotide&id=301785094) PREDICTED: Ailuropoda melanoleuca integrin, alpha 6 (ITGA6), mRNA

product length = 95

Forward primer 1 GTGACTGTGTTTCCCTCCAAG 21

Template 3106 .....C...........A... 3126

Reverse primer 1 GCAAGCATCAAAATCCCAAC 20

Template 3200 ..................G. 3181

[XM_003253716.2](http://www.ncbi.nlm.nih.gov/entrez/viewer.fcgi?db=nucleotide&id=441668079) PREDICTED: Nomascus leucogenys integrin, alpha 6, transcript variant 4 (ITGA6), mRNA

product length = 95

Forward primer 1 GTGACTGTGTTTCCCTCCAAG 21

Template 2908 .................A... 2928

Reverse primer 1 GCAAGCATCAAAATCCCAAC 20

Template 3002 ........T..G......G. 2983

[XM_003253714.2](http://www.ncbi.nlm.nih.gov/entrez/viewer.fcgi?db=nucleotide&id=441668077) PREDICTED: Nomascus leucogenys integrin, alpha 6, transcript variant 2 (ITGA6), mRNA

product length = 95

Forward primer 1 GTGACTGTGTTTCCCTCCAAG 21

Template 3223 .................A... 3243

Reverse primer 1 GCAAGCATCAAAATCCCAAC 20

Template 3317 ........T..G......G. 3298

[XM_003253713.2](http://www.ncbi.nlm.nih.gov/entrez/viewer.fcgi?db=nucleotide&id=441668074) PREDICTED: Nomascus leucogenys integrin, alpha 6, transcript variant 1 (ITGA6), mRNA

product length = 95

Forward primer 1 GTGACTGTGTTTCCCTCCAAG 21

Template 3223 .................A... 3243

Reverse primer 1 GCAAGCATCAAAATCCCAAC 20

Template 3317 ........T..G......G. 3298

[XM_003478536.1](http://www.ncbi.nlm.nih.gov/entrez/viewer.fcgi?db=nucleotide&id=348585649) PREDICTED: Cavia porcellus integrin alpha-6-like (LOC100731242), mRNA

product length = 95

Forward primer 1 GTGACTGTGTTTCCCTCCAAG 21

Template 3196 .................A... 3216

Reverse primer 1 GCAAGCATCAAAATCCCAAC 20

Template 3290 ..........GT......G. 3271

[XM_003406222.1](http://www.ncbi.nlm.nih.gov/entrez/viewer.fcgi?db=nucleotide&id=344268851) PREDICTED: Loxodonta africana integrin, alpha 6 (ITGA6), mRNA

product length = 95

Forward primer 1 GTGACTGTGTTTCCCTCCAAG 21

Template 2995 ........A............ 3015

Reverse primer 1 GCAAGCATCAAAATCCCAAC 20

Template 3089 C.............T...G. 3070

[XM_003495959.1](http://www.ncbi.nlm.nih.gov/entrez/viewer.fcgi?db=nucleotide&id=354467095) PREDICTED: Cricetulus griseus integrin alpha 6 (Itga6), mRNA

product length = 95

Forward primer 1 GTGACTGTGTTTCCCTCCAAG 21

Template 3169 .....A...........A... 3189

Reverse primer 1 GCAAGCATCAAAATCCCAAC 20

Template 3263 ..C...............G. 3244

**Target templates; primers for ITGB1**

[XM_002721189.1](http://www.ncbi.nlm.nih.gov/entrez/viewer.fcgi?db=nucleotide&id=291409904) PREDICTED: Oryctolagus cuniculus integrin, beta 1 (fibronectin receptor, beta polypeptide, antigen CD29 includes MDF2, MSK12) (ITGB1), mRNA

product length = 85

Forward primer 1 TGTAATGGCCGGGGTATCT 19

Template 1819 ................... 1837

Reverse primer 1 TCTGGCACATCTCACAGGTT 20

Template 1903 .................... 1884

[NM_133376.2](http://www.ncbi.nlm.nih.gov/entrez/viewer.fcgi?db=nucleotide&id=182507162) Homo sapiens integrin, beta 1 (fibronectin receptor, beta polypeptide, antigen CD29 includes MDF2, MSK12) (ITGB1), transcript variant 1E, mRNA

product length = 85

Forward primer 1 TGTAATGGCCGGGGTATCT 19

Template 1955 ..C...........C.... 1973

Reverse primer 1 TCTGGCACATCTCACAGGTT 20

Template 2039 ....A...........C... 2020

[NM_033668.2](http://www.ncbi.nlm.nih.gov/entrez/viewer.fcgi?db=nucleotide&id=182507160) Homo sapiens integrin, beta 1 (fibronectin receptor, beta polypeptide, antigen CD29 includes MDF2, MSK12) (ITGB1), transcript variant 1D, mRNA

product length = 85

Forward primer 1 TGTAATGGCCGGGGTATCT 19

Template 1819 ..C...........C.... 1837

Reverse primer 1 TCTGGCACATCTCACAGGTT 20

Template 1903 ....A...........C... 1884

[NM_001131852.1](http://www.ncbi.nlm.nih.gov/entrez/viewer.fcgi?db=nucleotide&id=197098821) Pongo abelii integrin, beta 1 (fibronectin receptor, beta polypeptide, antigen CD29 includes MDF2, MSK12) (ITGB1), mRNA

product length = 85

Forward primer 1 TGTAATGGCCGGGGTATCT 19

Template 1892 ..C...........C.... 1910

Reverse primer 1 TCTGGCACATCTCACAGGTT 20

Template 1976 ....A...........C... 1957

[NM_213968.1](http://www.ncbi.nlm.nih.gov/entrez/viewer.fcgi?db=nucleotide&id=47522763) Sus scrofa integrin, beta 1 (fibronectin receptor, beta polypeptide, antigen CD29 includes MDF2, MSK12) (ITGB1), mRNA

product length = 85

Forward primer 1 TGTAATGGCCGGGGTATCT 19

Template 2059 ..C............G... 2077

Reverse primer 1 TCTGGCACATCTCACAGGTT 20

Template 2143 ....A...........A... 2124

[NM_017022.2](http://www.ncbi.nlm.nih.gov/entrez/viewer.fcgi?db=nucleotide&id=158303323) Rattus norvegicus integrin, beta 1 (Itgb1), mRNA

product length = 85

Forward primer 1 TGTAATGGCCGGGGTATCT 19

Template 1989 ..C...........C.... 2007

Reverse primer 1 TCTGGCACATCTCACAGGTT 20

Template 2073 ....A..GG........... 2054

[XM_003364340.1](http://www.ncbi.nlm.nih.gov/entrez/viewer.fcgi?db=nucleotide&id=338721526) PREDICTED: Equus caballus integrin, beta 1 (fibronectin receptor, beta polypeptide, antigen CD29 includes MDF2, MSK12), transcript variant 2 (ITGB1), mRNA

product length = 85

Forward primer 1 TGTAATGGCCGGGGTATCT 19

Template 1819 ..C...........C.... 1837

Reverse primer 1 TCTGGCACATCTCACAGGTT 20

Template 1903 ....A............... 1884

[XM_001492665.3](http://www.ncbi.nlm.nih.gov/entrez/viewer.fcgi?db=nucleotide&id=338721525) PREDICTED: Equus caballus integrin, beta 1 (fibronectin receptor, beta polypeptide, antigen CD29 includes MDF2, MSK12), transcript variant 1 (ITGB1), mRNA

product length = 85

Forward primer 1 TGTAATGGCCGGGGTATCT 19

Template 1883 ..C...........C.... 1901

Reverse primer 1 TCTGGCACATCTCACAGGTT 20

Template 1967 ....A............... 1948

[XM_003903533.1](http://www.ncbi.nlm.nih.gov/entrez/viewer.fcgi?db=nucleotide&id=402879949) PREDICTED: Papio anubis integrin, beta 1 (fibronectin receptor, beta polypeptide, antigen CD29 includes MDF2, MSK12), transcript variant 2 (ITGB1), mRNA

product length = 85

Forward primer 1 TGTAATGGCCGGGGTATCT 19

Template 1949 ..............C.... 1967

Reverse primer 1 TCTGGCACATCTCACAGGTT 20

Template 2033 ....A...........T... 2014

[XM_003903532.1](http://www.ncbi.nlm.nih.gov/entrez/viewer.fcgi?db=nucleotide&id=402879947) PREDICTED: Papio anubis integrin, beta 1 (fibronectin receptor, beta polypeptide, antigen CD29 includes MDF2, MSK12), transcript variant 1 (ITGB1), mRNA

product length = 85

Forward primer 1 TGTAATGGCCGGGGTATCT 19

Template 2048 ..............C.... 2066

Reverse primer 1 TCTGGCACATCTCACAGGTT 20

Template 2132 ....A...........T... 2113

[XM_002805609.1](http://www.ncbi.nlm.nih.gov/entrez/viewer.fcgi?db=nucleotide&id=297300767) PREDICTED: Macaca mulatta integrin, beta 1 (fibronectin receptor, beta polypeptide, antigen CD29 includes MDF2, MSK12) (ITGB1), mRNA

product length = 85

Forward primer 1 TGTAATGGCCGGGGTATCT 19

Template 1937 ..............C.... 1955

Reverse primer 1 TCTGGCACATCTCACAGGTT 20

Template 2021 ....A...........T... 2002

[XM_004049247.1](http://www.ncbi.nlm.nih.gov/entrez/viewer.fcgi?db=nucleotide&id=426364389) PREDICTED: Gorilla gorilla gorilla integrin, beta 1 (fibronectin receptor, beta polypeptide, antigen CD29 includes MDF2, MSK12) (ITGB1), mRNA

product length = 85

Forward primer 1 TGTAATGGCCGGGGTATCT 19

Template 1949 ..C...........C.... 1967

Reverse primer 1 TCTGGCACATCTCACAGGTT 20

Template 2033 ....A...........C... 2014

[XM_003814791.1](http://www.ncbi.nlm.nih.gov/entrez/viewer.fcgi?db=nucleotide&id=397487506) PREDICTED: Pan paniscus integrin, beta 1 (fibronectin receptor, beta polypeptide, antigen CD29 includes MDF2, MSK12) (ITGB1), mRNA

product length = 85

Forward primer 1 TGTAATGGCCGGGGTATCT 19

Template 1821 ..C...........C.... 1839

Reverse primer 1 TCTGGCACATCTCACAGGTT 20

Template 1905 ....A...........C... 1886

[XM_507735.3](http://www.ncbi.nlm.nih.gov/entrez/viewer.fcgi?db=nucleotide&id=332833906) PREDICTED: Pan troglodytes integrin, beta 1 (fibronectin receptor, beta polypeptide, antigen CD29 includes MDF2, MSK12), transcript variant 2 (ITGB1), mRNA

product length = 85

Forward primer 1 TGTAATGGCCGGGGTATCT 19

Template 1949 ..C...........C.... 1967

Reverse primer 1 TCTGGCACATCTCACAGGTT 20

Template 2033 ....A...........C... 2014

[XM_003312513.1](http://www.ncbi.nlm.nih.gov/entrez/viewer.fcgi?db=nucleotide&id=332833904) PREDICTED: Pan troglodytes integrin, beta 1 (fibronectin receptor, beta polypeptide, antigen CD29 includes MDF2, MSK12), transcript variant 1 (ITGB1), mRNA

product length = 85

Forward primer 1 TGTAATGGCCGGGGTATCT 19

Template 1936 ..C...........C.... 1954

Reverse primer 1 TCTGGCACATCTCACAGGTT 20

Template 2020 ....A...........C... 2001

[XM_003276016.1](http://www.ncbi.nlm.nih.gov/entrez/viewer.fcgi?db=nucleotide&id=332253885) PREDICTED: Nomascus leucogenys integrin, beta 1 (fibronectin receptor, beta polypeptide, antigen CD29 includes MDF2, MSK12), transcript variant 3 (ITGB1), mRNA

product length = 85

Forward primer 1 TGTAATGGCCGGGGTATCT 19

Template 1956 ..C...........C.... 1974

Reverse primer 1 TCTGGCACATCTCACAGGTT 20

Template 2040 ....A...........T... 2021

[XM_003276015.1](http://www.ncbi.nlm.nih.gov/entrez/viewer.fcgi?db=nucleotide&id=332253883) PREDICTED: Nomascus leucogenys integrin, beta 1 (fibronectin receptor, beta polypeptide, antigen CD29 includes MDF2, MSK12), transcript variant 2 (ITGB1), mRNA

product length = 85

Forward primer 1 TGTAATGGCCGGGGTATCT 19

Template 2043 ..C...........C.... 2061

Reverse primer 1 TCTGGCACATCTCACAGGTT 20

Template 2127 ....A...........T... 2108

[XM_003276014.1](http://www.ncbi.nlm.nih.gov/entrez/viewer.fcgi?db=nucleotide&id=332253881) PREDICTED: Nomascus leucogenys integrin, beta 1 (fibronectin receptor, beta polypeptide, antigen CD29 includes MDF2, MSK12), transcript variant 1 (ITGB1), mRNA

product length = 85

Forward primer 1 TGTAATGGCCGGGGTATCT 19

Template 2049 ..C...........C.... 2067

Reverse primer 1 TCTGGCACATCTCACAGGTT 20

Template 2133 ....A...........T... 2114

[NM_002211.3](http://www.ncbi.nlm.nih.gov/entrez/viewer.fcgi?db=nucleotide&id=182519230) Homo sapiens integrin, beta 1 (fibronectin receptor, beta polypeptide, antigen CD29 includes MDF2, MSK12) (ITGB1), transcript variant 1A, mRNA

product length = 85

Forward primer 1 TGTAATGGCCGGGGTATCT 19

Template 2040 ..C...........C.... 2058

Reverse primer 1 TCTGGCACATCTCACAGGTT 20

Template 2124 ....A...........C... 2105

[XM_001508152.1](http://www.ncbi.nlm.nih.gov/entrez/viewer.fcgi?db=nucleotide&id=149634687) PREDICTED: Ornithorhynchus anatinus integrin beta-1-like, transcript variant 2 (LOC100073959), mRNA

product length = 85

Forward primer 1 TGTAATGGCCGGGGTATCT 19

Template 1822 ..C........C..G.... 1840

Reverse primer 1 TCTGGCACATCTCACAGGTT 20

Template 1906 ....A..............C 1887

[XM_003508410.1](http://www.ncbi.nlm.nih.gov/entrez/viewer.fcgi?db=nucleotide&id=354492645) PREDICTED: Cricetulus griseus integrin beta 1 (fibronectin receptor beta) (Itgb1), mRNA

product length = 85

Forward primer 1 TGTAATGGCCGGGGTATCT 19

Template 1823 ..C............G... 1841

Reverse primer 1 TCTGGCACATCTCACAGGTT 20

Template 1907 ....A..TG........... 1888

**Target templates; primers for MMP2**

[NM_001082209.1](http://www.ncbi.nlm.nih.gov/entrez/viewer.fcgi?db=nucleotide&id=126722976) Oryctolagus cuniculus matrix metallopeptidase 2 (gelatinase A, 72kDa gelatinase, 72kDa type IV collagenase) (MMP2), mRNA

product length = 90

Forward primer 1 CCCCAAAACGGACAAAGAG 19

Template 126 ................... 144

Reverse primer 1 TCCTTCAGCACGAACAGGT 19

Template 215 ................... 197

[NM_214192.1](http://www.ncbi.nlm.nih.gov/entrez/viewer.fcgi?db=nucleotide&id=47523461) Sus scrofa matrix metallopeptidase 2 (gelatinase A, 72kDa gelatinase, 72kDa type IV collagenase) (MMP2), mRNA

product length = 90

Forward primer 1 CCCCAAAACGGACAAAGAG 19

Template 177 ................... 195

Reverse primer 1 TCCTTCAGCACGAACAGGT 19

Template 266 ................... 248

[NM_004530.4](http://www.ncbi.nlm.nih.gov/entrez/viewer.fcgi?db=nucleotide&id=189217851) Homo sapiens matrix metallopeptidase 2 (gelatinase A, 72kDa gelatinase, 72kDa type IV collagenase) (MMP2), transcript variant 1, mRNA

product length = 90

Forward primer 1 CCCCAAAACGGACAAAGAG 19

Template 437 ................... 455

Reverse primer 1 TCCTTCAGCACGAACAGGT 19

Template 526 ...........A....... 508

[NM_001166180.1](http://www.ncbi.nlm.nih.gov/entrez/viewer.fcgi?db=nucleotide&id=261244993) Ovis aries matrix metallopeptidase 2 (gelatinase A, 72kDa gelatinase, 72kDa type IV collagenase) (MMP2), mRNA

product length = 90

Forward primer 1 CCCCAAAACGGACAAAGAG 19

Template 129 ................... 147

Reverse primer 1 TCCTTCAGCACGAACAGGT 19

Template 218 ...........A....A.. 200

[NM_174745.2](http://www.ncbi.nlm.nih.gov/entrez/viewer.fcgi?db=nucleotide&id=31340835) Bos taurus matrix metallopeptidase 2 (gelatinase A, 72kDa gelatinase, 72kDa type IV collagenase) (MMP2), mRNA

product length = 90

Forward primer 1 CCCCAAAACGGACAAAGAG 19

Template 310 ................... 328

Reverse primer 1 TCCTTCAGCACGAACAGGT 19

Template 399 ...........A....A.. 381

[NM_031054.2](http://www.ncbi.nlm.nih.gov/entrez/viewer.fcgi?db=nucleotide&id=146262018) Rattus norvegicus matrix metallopeptidase 2 (Mmp2), mRNA

product length = 90

Forward primer 1 CCCCAAAACGGACAAAGAG 19

Template 417 .........A......... 435

Reverse primer 1 TCCTTCAGCACGAACAGGT 19

Template 506 ...........A..G.... 488

[XM_003263057.2](http://www.ncbi.nlm.nih.gov/entrez/viewer.fcgi?db=nucleotide&id=441597247) PREDICTED: Nomascus leucogenys matrix metallopeptidase 2 (gelatinase A, 72kDa gelatinase, 72kDa type IV collagenase) (MMP2), mRNA

product length = 90

Forward primer 1 CCCCAAAACGGACAAAGAG 19

Template 438 ................... 456

Reverse primer 1 TCCTTCAGCACGAACAGGT 19

Template 527 ...........A....... 509

[XM_004057657.1](http://www.ncbi.nlm.nih.gov/entrez/viewer.fcgi?db=nucleotide&id=426382207) PREDICTED: Gorilla gorilla gorilla matrix metallopeptidase 2 (gelatinase A, 72kDa gelatinase, 72kDa type IV collagenase), transcript variant 1 (MMP2), mRNA

product length = 90

Forward primer 1 CCCCAAAACGGACAAAGAG 19

Template 662 ................... 680

Reverse primer 1 TCCTTCAGCACGAACAGGT 19

Template 751 ...........A....... 733

[XM_003937277.1](http://www.ncbi.nlm.nih.gov/entrez/viewer.fcgi?db=nucleotide&id=403292605) PREDICTED: Saimiri boliviensis boliviensis matrix metallopeptidase 2 (gelatinase A, 72kDa gelatinase, 72kDa type IV collagenase) (MMP2), mRNA

product length = 90

Forward primer 1 CCCCAAAACGGACAAAGAG 19

Template 647 ................... 665

Reverse primer 1 TCCTTCAGCACGAACAGGT 19

Template 736 .................A. 718

[XM_002826433.2](http://www.ncbi.nlm.nih.gov/entrez/viewer.fcgi?db=nucleotide&id=395747845) PREDICTED: Pongo abelii matrix metallopeptidase 2 (gelatinase A, 72kDa gelatinase, 72kDa type IV collagenase) (MMP2), mRNA

product length = 90

Forward primer 1 CCCCAAAACGGACAAAGAG 19

Template 263 ................... 281

Reverse primer 1 TCCTTCAGCACGAACAGGT 19

Template 352 ...........A....... 334

[XM_002761017.2](http://www.ncbi.nlm.nih.gov/entrez/viewer.fcgi?db=nucleotide&id=390477709) PREDICTED: Callithrix jacchus matrix metallopeptidase 2 (gelatinase A, 72kDa gelatinase, 72kDa type IV collagenase) (MMP2), mRNA

product length = 90

Forward primer 1 CCCCAAAACGGACAAAGAG 19

Template 126 ...A............... 144

Reverse primer 1 TCCTTCAGCACGAACAGGT 19

Template 215 ................... 197

[XM_003416312.1](http://www.ncbi.nlm.nih.gov/entrez/viewer.fcgi?db=nucleotide&id=344289254) PREDICTED: Loxodonta africana matrix metallopeptidase 2 (gelatinase A, 72kDa gelatinase, 72kDa type IV collagenase) (MMP2), mRNA

product length = 90

Forward primer 1 CCCCAAAACGGACAAAGAG 19

Template 126 ................... 144

Reverse primer 1 TCCTTCAGCACGAACAGGT 19

Template 215 ...........A....... 197

[XM_001167520.2](http://www.ncbi.nlm.nih.gov/entrez/viewer.fcgi?db=nucleotide&id=332845944) PREDICTED: Pan troglodytes matrix metallopeptidase 2 (gelatinase A, 72kDa gelatinase, 72kDa type IV collagenase), transcript variant 4 (MMP2), mRNA

product length = 90

Forward primer 1 CCCCAAAACGGACAAAGAG 19

Template 126 ................... 144

Reverse primer 1 TCCTTCAGCACGAACAGGT 19

Template 215 ...........A....... 197

[XM_001087939.2](http://www.ncbi.nlm.nih.gov/entrez/viewer.fcgi?db=nucleotide&id=297284002) PREDICTED: Macaca mulatta matrix metallopeptidase 2 (gelatinase A, 72kDa gelatinase, 72kDa type IV collagenase), transcript variant 4 (MMP2), mRNA

product length = 90

Forward primer 1 CCCCAAAACGGACAAAGAG 19

Template 257 ................... 275

Reverse primer 1 TCCTTCAGCACGAACAGGT 19

Template 346 ...........A....... 328

[XM_003916886.1](http://www.ncbi.nlm.nih.gov/entrez/viewer.fcgi?db=nucleotide&id=402908410) PREDICTED: Papio anubis matrix metallopeptidase 2 (gelatinase A, 72kDa gelatinase, 72kDa type IV collagenase) (MMP2), partial mRNA

product length = 90

Forward primer 1 CCCCAAAACGGACAAAGAG 19

Template 258 ................... 276

Reverse primer 1 TCCTTCAGCACGAACAGGT 19

Template 347 ........A..A....... 329

[XM_003477541.1](http://www.ncbi.nlm.nih.gov/entrez/viewer.fcgi?db=nucleotide&id=348583656) PREDICTED: Cavia porcellus 72 kDa type IV collagenase-like (LOC100719361), mRNA

product length = 90

Forward primer 1 CCCCAAAACGGACAAAGAG 19

Template 126 T.................. 144

Reverse primer 1 TCCTTCAGCACGAACAGGT 19

Template 215 ...........A....... 197

[XM_003998042.1](http://www.ncbi.nlm.nih.gov/entrez/viewer.fcgi?db=nucleotide&id=410983528) PREDICTED: Felis catus matrix metallopeptidase 2 (gelatinase A, 72kDa gelatinase, 72kDa type IV collagenase) (MMP2), mRNA

product length = 90

Forward primer 1 CCCCAAAACGGACAAAGAG 19

Template 7 ...A............... 25

Reverse primer 1 TCCTTCAGCACGAACAGGT 19

Template 96 .....G............. 78

[XM_003792545.1](http://www.ncbi.nlm.nih.gov/entrez/viewer.fcgi?db=nucleotide&id=395839431) PREDICTED: Otolemur garnettii matrix metallopeptidase 2 (gelatinase A, 72kDa gelatinase, 72kDa type IV collagenase) (MMP2), mRNA

product length = 90

Forward primer 1 CCCCAAAACGGACAAAGAG 19

Template 476 .........A......... 494

Reverse primer 1 TCCTTCAGCACGAACAGGT 19

Template 565 ...........A....... 547

[XM_003757257.1](http://www.ncbi.nlm.nih.gov/entrez/viewer.fcgi?db=nucleotide&id=395505961) PREDICTED: Sarcophilus harrisii matrix metallopeptidase 2 (gelatinase A, 72kDa gelatinase, 72kDa type IV collagenase) (MMP2), mRNA

product length = 90

Forward primer 1 CCCCAAAACGGACAAAGAG 19

Template 161 T........A......... 179

Reverse primer 1 TCCTTCAGCACGAACAGGT 19

Template 250 ...........A....... 232

[XM_001372993.2](http://www.ncbi.nlm.nih.gov/entrez/viewer.fcgi?db=nucleotide&id=334311809) PREDICTED: Monodelphis domestica 72 kDa type IV collagenase-like (LOC100020585), mRNA

product length = 90

Forward primer 1 CCCCAAAACGGACAAAGAG 19

Template 126 T........A......... 144

Reverse primer 1 TCCTTCAGCACGAACAGGT 19

Template 215 .......A...A....... 197

[NM_008610.2](http://www.ncbi.nlm.nih.gov/entrez/viewer.fcgi?db=nucleotide&id=47271505) Mus musculus matrix metallopeptidase 2 (Mmp2), mRNA

product length = 90

Forward primer 1 CCCCAAAACGGACAAAGAG 19

Template 423 ...T.....A......... 441

Reverse primer 1 TCCTTCAGCACGAACAGGT 19

Template 512 ..T........A..G.... 494

**Target templates; primers for MMP14**

The primers for MMP14 reported in the current manuscript were designed based on the known human sequence and similarities with other species, as the rabbit sequence was not determined.

[NM_004995.2](http://www.ncbi.nlm.nih.gov/entrez/viewer.fcgi?db=nucleotide&id=13027797) Homo sapiens matrix metallopeptidase 14 (MMP14), mRNA

product length = 131

Forward primer 1 CGAGGGGAGATGTTTGTCTT 20

Template 1222 .................... 1241

Reverse primer 1 TCGTAGGCAGTGTTGATGGA 20

Template 1352 .................... 1333

[NM_001131321.2](http://www.ncbi.nlm.nih.gov/entrez/viewer.fcgi?db=nucleotide&id=402743735) Pongo abelii matrix metallopeptidase 14 (MMP14), mRNA

product length = 131

Forward primer 1 CGAGGGGAGATGTTTGTCTT 20

Template 1262 .................... 1281

Reverse primer 1 TCGTAGGCAGTGTTGATGGA 20

Template 1392 .................... 1373

[NM_001266810.1](http://www.ncbi.nlm.nih.gov/entrez/viewer.fcgi?db=nucleotide&id=388453116) Macaca mulatta matrix metallopeptidase 14 (MMP14), mRNA

product length = 131

Forward primer 1 CGAGGGGAGATGTTTGTCTT 20

Template 1223 .................... 1242

Reverse primer 1 TCGTAGGCAGTGTTGATGGA 20

Template 1353 ..A................. 1334

[NM_174390.2](http://www.ncbi.nlm.nih.gov/entrez/viewer.fcgi?db=nucleotide&id=31342134) Bos taurus matrix metallopeptidase 14 (MMP14), mRNA

product length = 131

Forward primer 1 CGAGGGGAGATGTTTGTCTT 20

Template 1041 .................... 1060

Reverse primer 1 TCGTAGGCAGTGTTGATGGA 20

Template 1171 ..A................. 1152

[NM_214239.1](http://www.ncbi.nlm.nih.gov/entrez/viewer.fcgi?db=nucleotide&id=47523559) Sus scrofa matrix metallopeptidase 14 (MMP14), mRNA

product length = 131

Forward primer 1 CGAGGGGAGATGTTTGTCTT 20

Template 982 .................T.. 1001

Reverse primer 1 TCGTAGGCAGTGTTGATGGA 20

Template 1112 .................... 1093

[NM_008608.3](http://www.ncbi.nlm.nih.gov/entrez/viewer.fcgi?db=nucleotide&id=188528636) Mus musculus matrix metallopeptidase 14 (Mmp14), mRNA

product length = 131

Forward primer 1 CGAGGGGAGATGTTTGTCTT 20

Template 1250 .....A.............. 1269

Reverse primer 1 TCGTAGGCAGTGTTGATGGA 20

Template 1380 ...........A........ 1361

[NM_001166181.1](http://www.ncbi.nlm.nih.gov/entrez/viewer.fcgi?db=nucleotide&id=261244995) Ovis aries matrix metallopeptidase 14 (MMP14), mRNA

product length = 131

Forward primer 1 CGAGGGGAGATGTTTGTCTT 20

Template 985 .................... 1004

Reverse primer 1 TCGTAGGCAGTGTTGATGGA 20

Template 1115 ..A................. 1096

[NM_031056.1](http://www.ncbi.nlm.nih.gov/entrez/viewer.fcgi?db=nucleotide&id=13591994) Rattus norvegicus matrix metallopeptidase 14 (Mmp14), mRNA

product length = 131

Forward primer 1 CGAGGGGAGATGTTTGTCTT 20

Template 1151 .....A.............. 1170

Reverse primer 1 TCGTAGGCAGTGTTGATGGA 20

Template 1281 ..A................. 1262

[XM_003260581.2](http://www.ncbi.nlm.nih.gov/entrez/viewer.fcgi?db=nucleotide&id=441667185) PREDICTED: Nomascus leucogenys matrix metallopeptidase 14 (MMP14), mRNA

product length = 131

Forward primer 1 CGAGGGGAGATGTTTGTCTT 20

Template 1272 .................... 1291

Reverse primer 1 TCGTAGGCAGTGTTGATGGA 20

Template 1402 .................... 1383

[XM_001157686.3](http://www.ncbi.nlm.nih.gov/entrez/viewer.fcgi?db=nucleotide&id=410048006) PREDICTED: Pan troglodytes matrix metallopeptidase 14, transcript variant 4 (MMP14), mRNA

product length = 131

Forward primer 1 CGAGGGGAGATGTTTGTCTT 20

Template 1272 .................... 1291

Reverse primer 1 TCGTAGGCAGTGTTGATGGA 20

Template 1402 .................... 1383

[XM_003808123.1](http://www.ncbi.nlm.nih.gov/entrez/viewer.fcgi?db=nucleotide&id=397473336) PREDICTED: Pan paniscus matrix metallopeptidase 14 (MMP14), mRNA

product length = 131

Forward primer 1 CGAGGGGAGATGTTTGTCTT 20

Template 1272 .................... 1291

Reverse primer 1 TCGTAGGCAGTGTTGATGGA 20

Template 1402 .................... 1383

[XM_851854.2](http://www.ncbi.nlm.nih.gov/entrez/viewer.fcgi?db=nucleotide&id=345804004) PREDICTED: Canis lupus familiaris matrix metallopeptidase 14, transcript variant 2 (MMP14), mRNA

product length = 131

Forward primer 1 CGAGGGGAGATGTTTGTCTT 20

Template 1199 .................... 1218

Reverse primer 1 TCGTAGGCAGTGTTGATGGA 20

Template 1329 .................... 1310

[XM_002925997.1](http://www.ncbi.nlm.nih.gov/entrez/viewer.fcgi?db=nucleotide&id=301781261) PREDICTED: Ailuropoda melanoleuca matrix metalloproteinase-14-like (LOC100468621), mRNA

product length = 131

Forward primer 1 CGAGGGGAGATGTTTGTCTT 20

Template 1130 .................... 1149

Reverse primer 1 TCGTAGGCAGTGTTGATGGA 20

Template 1260 .................... 1241

[XM_004054975.1](http://www.ncbi.nlm.nih.gov/entrez/viewer.fcgi?db=nucleotide&id=426376470) PREDICTED: Gorilla gorilla gorilla matrix metallopeptidase 14 (MMP14), partial mRNA

product length = 131

Forward primer 1 CGAGGGGAGATGTTTGTCTT 20

Template 1272 .................T.. 1291

Reverse primer 1 TCGTAGGCAGTGTTGATGGA 20

Template 1402 .................... 1383

[XM_003987454.1](http://www.ncbi.nlm.nih.gov/entrez/viewer.fcgi?db=nucleotide&id=410961873) PREDICTED: Felis catus matrix metallopeptidase 14 (MMP14), mRNA

product length = 131

Forward primer 1 CGAGGGGAGATGTTTGTCTT 20

Template 1211 .................... 1230

Reverse primer 1 TCGTAGGCAGTGTTGATGGA 20

Template 1341 ..............A..... 1322

[XM_003924336.1](http://www.ncbi.nlm.nih.gov/entrez/viewer.fcgi?db=nucleotide&id=403264211) PREDICTED: Saimiri boliviensis boliviensis matrix metallopeptidase 14 (MMP14), mRNA

product length = 131

Forward primer 1 CGAGGGGAGATGTTTGTCTT 20

Template 1268 .................... 1287

Reverse primer 1 TCGTAGGCAGTGTTGATGGA 20

Template 1398 ..A................. 1379

[XM_003901563.1](http://www.ncbi.nlm.nih.gov/entrez/viewer.fcgi?db=nucleotide&id=402875651) PREDICTED: Papio anubis matrix metallopeptidase 14 (MMP14), mRNA

product length = 131

Forward primer 1 CGAGGGGAGATGTTTGTCTT 20

Template 1269 .................... 1288

Reverse primer 1 TCGTAGGCAGTGTTGATGGA 20

Template 1399 ..A................. 1380

[XM_002753610.2](http://www.ncbi.nlm.nih.gov/entrez/viewer.fcgi?db=nucleotide&id=390468850) PREDICTED: Callithrix jacchus matrix metallopeptidase 14 (MMP14), mRNA

product length = 131

Forward primer 1 CGAGGGGAGATGTTTGTCTT 20

Template 1168 .................... 1187

Reverse primer 1 TCGTAGGCAGTGTTGATGGA 20

Template 1298 ..A................. 1279

[XM_003420935.1](http://www.ncbi.nlm.nih.gov/entrez/viewer.fcgi?db=nucleotide&id=344298606) PREDICTED: Loxodonta africana matrix metallopeptidase 14 (MMP14), mRNA

product length = 131

Forward primer 1 CGAGGGGAGATGTTTGTCTT 20

Template 988 .................... 1007

Reverse primer 1 TCGTAGGCAGTGTTGATGGA 20

Template 1118 .................A.. 1099

[XM_001495103.3](http://www.ncbi.nlm.nih.gov/entrez/viewer.fcgi?db=nucleotide&id=338717682) PREDICTED: Equus caballus matrix metallopeptidase 14 (MMP14), mRNA

product length = 131

Forward primer 1 CGAGGGGAGATGTTTGTCTT 20

Template 1087 .................... 1106

Reverse primer 1 TCGTAGGCAGTGTTGATGGA 20

Template 1217 ..A................. 1198

[XM_003474502.1](http://www.ncbi.nlm.nih.gov/entrez/viewer.fcgi?db=nucleotide&id=348577556) PREDICTED: Cavia porcellus matrix metallopeptidase 14 (Mmp14), mRNA

product length = 131

Forward primer 1 CGAGGGGAGATGTTTGTCTT 20

Template 979 .................G.. 998

Reverse primer 1 TCGTAGGCAGTGTTGATGGA 20

Template 1109 ..A................. 1090

[XM_003801908.1](http://www.ncbi.nlm.nih.gov/entrez/viewer.fcgi?db=nucleotide&id=395859256) PREDICTED: Otolemur garnettii matrix metallopeptidase 14 (MMP14), mRNA

product length = 131

Forward primer 1 CGAGGGGAGATGTTTGTCTT 20

Template 1264 .....A.............. 1283

Reverse primer 1 TCGTAGGCAGTGTTGATGGA 20

Template 1394 ..A..C...........A.. 1375

[NM_001246769.1](http://www.ncbi.nlm.nih.gov/entrez/viewer.fcgi?db=nucleotide&id=350538020) Cricetulus griseus matrix metallopeptidase 14 (Mmp14), mRNA

product length = 131

Forward primer 1 CGAGGGGAGATGTTTGTCTT 20

Template 1166 .....A.............. 1185

Reverse primer 1 TCGTAGGCAGTGTTGATGGA 20

Template 1296 ..A........A..C..... 1277

[XM_003755864.1](http://www.ncbi.nlm.nih.gov/entrez/viewer.fcgi?db=nucleotide&id=395503101) PREDICTED: Sarcophilus harrisii matrix metallopeptidase 14 (MMP14), mRNA

product length = 131

Forward primer 1 CGAGGGGAGATGTTTGTCTT 20

Template 985 ..G..A..A........... 1004

Reverse primer 1 TCGTAGGCAGTGTTGATGGA 20

Template 1115 ........C........A.. 1096

[XM_003428662.1](http://www.ncbi.nlm.nih.gov/entrez/viewer.fcgi?db=nucleotide&id=345308552) PREDICTED: Ornithorhynchus anatinus matrix metalloproteinase-14-like (LOC100091029), partial mRNA

product length = 131

Forward primer 1 CGAGGGGAGATGTTTGTCTT 20

Template 982 ..T...........C..G.. 1001

Reverse primer 1 TCGTAGGCAGTGTTGATGGA 20

Template 1112 ..A.....G........... 1093

**Target templates; primers for TIMP1**

[NM_001082232.2](http://www.ncbi.nlm.nih.gov/entrez/viewer.fcgi?db=nucleotide&id=283806669) Oryctolagus cuniculus tissue inhibitor of metalloproteinase 1 (LOC100009047), mRNA

product length = 80

Forward primer 1 ACTGAAGGCTGCTCCTGTTG 20

Template 664 .................... 683

Reverse primer 1 GTGTGGGACAAAGAAAGATGG 21

Template 743 ..................... 723

**Target templates; primers for TIMP2**

[XM_002723776.1](http://www.ncbi.nlm.nih.gov/entrez/viewer.fcgi?db=nucleotide&id=291415159) PREDICTED: Oryctolagus cuniculus TIMP metallopeptidase inhibitor 2 (TIMP2), mRNA

product length = 102

Forward primer 1 CGACAAGGACATCGAGTTCA 20

Template 159 .................... 178

Reverse primer 1 GCCTTCCCTGCAATGAGATA 20

Template 260 .................... 241

[NM_001166186.1](http://www.ncbi.nlm.nih.gov/entrez/viewer.fcgi?db=nucleotide&id=261244947) Ovis aries TIMP metallopeptidase inhibitor 2 (TIMP2), mRNA

product length = 102

Forward primer 1 CGACAAGGACATCGAGTTCA 20

Template 246 T...C.......A.....T. 265

Reverse primer 1 GCCTTCCCTGCAATGAGATA 20

Template 347 .................... 328

[NM_001003082.1](http://www.ncbi.nlm.nih.gov/entrez/viewer.fcgi?db=nucleotide&id=50978759) Canis lupus familiaris TIMP metallopeptidase inhibitor 2 (TIMP2), mRNA

product length = 102

Forward primer 1 CGACAAGGACATCGAGTTCA 20

Template 248 A...........A.....T. 267

Reverse primer 1 GCCTTCCCTGCAATGAGATA 20

Template 349 .....T..C........... 330

[NM_011594.3](http://www.ncbi.nlm.nih.gov/entrez/viewer.fcgi?db=nucleotide&id=61835847) Mus musculus tissue inhibitor of metalloproteinase 2 (Timp2), mRNA

product length = 102

Forward primer 1 CGACAAGGACATCGAGTTCA 20

Template 567 T.....A...........T. 586

Reverse primer 1 GCCTTCCCTGCAATGAGATA 20

Template 668 .....T........T..... 649

[XM_003997248.1](http://www.ncbi.nlm.nih.gov/entrez/viewer.fcgi?db=nucleotide&id=410981889) PREDICTED: Felis catus TIMP metallopeptidase inhibitor 2 (TIMP2), mRNA

product length = 102

Forward primer 1 CGACAAGGACATCGAGTTCA 20

Template 264 T...........A.....T. 283

Reverse primer 1 GCCTTCCCTGCAATGAGATA 20

Template 365 .....T.............. 346

[XM_003417356.1](http://www.ncbi.nlm.nih.gov/entrez/viewer.fcgi?db=nucleotide&id=344291360) PREDICTED: Loxodonta africana metalloproteinase inhibitor 2-like (LOC100662033), mRNA

product length = 102

Forward primer 1 CGACAAGGACATCGAGTTCA 20

Template 465 T...........A.....T. 484

Reverse primer 1 GCCTTCCCTGCAATGAGATA 20

Template 566 .....A.............. 547

[XM_002918379.1](http://www.ncbi.nlm.nih.gov/entrez/viewer.fcgi?db=nucleotide&id=301766031) PREDICTED: Ailuropoda melanoleuca metalloproteinase inhibitor 2-like (LOC100476303), mRNA

product length = 102

Forward primer 1 CGACAAGGACATCGAGTTCA 20

Template 289 T...........A.....T. 308

Reverse primer 1 GCCTTCCCTGCAATGAGATA 20

Template 390 .....T.............. 371

[XM_003786083.1](http://www.ncbi.nlm.nih.gov/entrez/viewer.fcgi?db=nucleotide&id=395825844) PREDICTED: Otolemur garnettii TIMP metallopeptidase inhibitor 2 (TIMP2), mRNA

product length = 102

Forward primer 1 CGACAAGGACATCGAGTTCA 20

Template 505 T...........A....... 524

Reverse primer 1 GCCTTCCCTGCAATGAGATA 20

Template 606 ..T..T.............. 587

[XM_003931678.1](http://www.ncbi.nlm.nih.gov/entrez/viewer.fcgi?db=nucleotide&id=403280440) PREDICTED: Saimiri boliviensis boliviensis TIMP metallopeptidase inhibitor 2, transcript variant 2 (TIMP2), mRNA

product length = 102

Forward primer 1 CGACAAGGACATCGAGTTCA 20

Template 225 T..G........A.....T. 244

Reverse primer 1 GCCTTCCCTGCAATGAGATA 20

Template 326 .....T.............. 307

[XM_003931677.1](http://www.ncbi.nlm.nih.gov/entrez/viewer.fcgi?db=nucleotide&id=403280438) PREDICTED: Saimiri boliviensis boliviensis TIMP metallopeptidase inhibitor 2, transcript variant 1 (TIMP2), mRNA

product length = 102

Forward primer 1 CGACAAGGACATCGAGTTCA 20

Template 309 T..G........A.....T. 328

Reverse primer 1 GCCTTCCCTGCAATGAGATA 20

Template 410 .....T.............. 391

[XM_002748803.2](http://www.ncbi.nlm.nih.gov/entrez/viewer.fcgi?db=nucleotide&id=390463859) PREDICTED: Callithrix jacchus TIMP metallopeptidase inhibitor 2 (TIMP2), mRNA

product length = 102

Forward primer 1 CGACAAGGACATCGAGTTCA 20

Template 176 T..A........A.....T. 195

Reverse primer 1 GCCTTCCCTGCAATGAGATA 20

Template 277 .....T.............. 258

[XM_003498832.1](http://www.ncbi.nlm.nih.gov/entrez/viewer.fcgi?db=nucleotide&id=354473312) PREDICTED: Cricetulus griseus metalloproteinase inhibitor 2-like (LOC100772625), mRNA

product length = 102

Forward primer 1 CGACAAGGACATCGAGTTCA 20

Template 216 T.....A...........T. 235

Reverse primer 1 GCCTTCCCTGCAATGAGATA 20

Template 317 .....T........T..... 298
